# Polarized Color Filters Using Colloidal Quantum Rod Nanocrystals for Advanced High‐Performance Displays

**DOI:** 10.1002/advs.202414316

**Published:** 2025-04-09

**Authors:** Jianxin Song, Maksym F. Prodanov, Yiyang Gao, Chengbin Kang, Debjyoti Bhadra, Yuechu Cheng, Zebing Liao, Kumar Mallem, Vigneshwaran Swaminathan, Valerii V. Vashchenko, Xiao Wu, Xinhui Lu, Abhishek K. Srivastava

**Affiliations:** ^1^ State Key Laboratory of Advanced Displays and Optoelectronics Technologies Department of Electronics and Computer Engineering The Hong Kong University of Science and Technology Clear Water Bay Hong Kong 999077 China; ^2^ Centre for Display Research Department of Electronics and Computer Engineering The Hong Kong University of Science and Technology Clear Water Bay Hong Kong 999077 China; ^3^ IAS Center for Quantum Technologies The Hong Kong University of Science and Technology Clear Water Bay Hong Kong 999077 China; ^4^ Department of Physics The Chinese University of Hong Kong Hong Kong 999077 China

**Keywords:** alignment, photolithography, photoluminescent color filters, polarized emission, quantum rods

## Abstract

In this article, color filters (CFs) emitting linearly polarized light, achieving both full‐color and pixel‐level precision, which can simultaneously offer high color saturation, high ambient contrast ratio (ACR), and high efficiency for modern displays, are disclosed. The quantum rods (QRs) exhibit a narrow‐band polarized emission that effectively mitigates traditional challenges of color purity and light losses. Specially designed ligands on the QR surfaces allow higher load of the QR material into the polymer matrix with uniform alignment. Optimizing inkjet printing, photolithography, and photoalignment processes, a remarkable degree of polarization (DOP) of 0.65 is achieved for precisely patterned red and green QR CFs. Due to the polarized emission of unidirectionally aligned QRs, the liquid crystal display (LCD) equipped with the designed QRCF shows enhanced efficiency and ACR, highlighting advantages for their application in next‐generation displays over other types of photoluminescence CFs.

## Graphical abstract

This groundbreaking research has advanced the utilization of quantum rods (QRs) for the emission of linearly polarized light, resulting in exceptional color saturation and enhanced display efficiency. The developed filter demonstrates a polarization degree of up to 0.67 and an impressive ambient contrast ratio (ACR) of 263:1, setting the stage for significant performance enhancements in next‐generation display technologies.

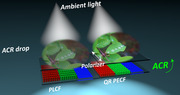

## 1 Introduction

Full‐color display technology, a foundational pillar of visual technology, has seen a dynamic and multifaceted evolution in recent times. This technology leverages color as a medium to translate the complexity and richness of the tangible world into visual information. The Liquid Crystal Displays (LCD), known for their reliability, ease of integration, and aesthetic appearance, have become the predominant display technology. Subsequently, OLED technology, characterized by exceptional contrast and design flexibility, has initiated a novel era in the chronicle of display technology.^[^ ^1^ ^]^ Though the image quality has improved significantly in the past several years, reaching the limits of human vision, these displays' efficiency is still struggling in the range of 3–5%, which further drops for the displays with high pixel densities. The Energy Efficiency Index (*EEI*) measures the power consumption for the display products, which is defined as: *EEI*   =  (*P_measured_* + 1)/(3*[90*tanh(0.02 + 0.004*(*A* − 11) + 4)] + 3 + *corr*). Here, *P_measured_* is the measured power in Watts in ON mode in the normal configuration (in standard dynamic range), and *corr* is a correction factor. The European Union, on 1st March 2023, revised the EEI limit for modern display in ON mode to 0.9. The EEI of commercially available displays ranges between 1.4 and 1.8. One of the critical components in displays (both LCD and OLEDs) is a color filter (CF). Its absorptive nature induces more than 70% of optical losses,^[^ ^2^ ^]^ making it difficult to further improve display energy efficiency. Extending battery life requires sacrifices in display brightness, image frequency, or other performance aspects.

The white LED light from the backlight, in traditional LCDs, passes through the polarizer and then the liquid crystal cell to control the brightness and final colors. The colors in traditional LCD panels are primarily obtained through organic dyes, which absorb light in certain wavelength regions. Three types (Red, Green, and Blue (RGB)) of absorptive CFs (ACFs) in each pixel provide full‐color images but compromise optical efficiency due to most of the initial white light being absorbed. Furthermore, due to the wider absorption band and significant overlap among R, G, and B absorption bands, the ACFs show poor color saturation. The same is true for the white OLEDs, which also use the ACFs for color reproductions.

Recently, CF fabricated using photoluminescent (PL) materials, also known as PLCFs, are attracting increasing attention due to the realization of the full‐color emissive type of display with higher brightness, better efficiency, larger viewing angle, and enabling access to the micro‐LED display sector.^[^ ^3^ ^]^ Replacing ACFs with PLCF and using short‐wavelength (blue/UV) backlight in LCD improves efficiency and color performance, and this technology has a particularly far‐reaching impact within the display industry.^[^ ^3^ ^]^ The LCD equipped with the PLCFs shows high brightness and a large color gamut.

In contrast, fluorescent organic dyes‐based PLCFs face issues such as limited internal quantum efficiency (IQE) due to aggregation‐caused quenching phenomena and low color purity owing to broad emission spectra.^[^ ^1^, ^4^ ^]^ Although efforts have been made to mitigate these problems, such as molecular chemical modifications and encapsulation to suppress aggregation^[^ ^5^ ^]^ or synthesizing materials into organic quantum dots and utilizing micro‐cavity structures to reduce the emission bandwidth,^[^ ^6^ ^]^ these approaches substantially increase the complexity and cost of CF fabrication.^[^ ^7^ ^]^ Recently, the emergence of Mini/Micro LED display technology has come to the forefront, marking a pivotal development in next‐generation display technology.^[^ ^8^ ^]^ With its benefits of high resolution, broad color gamut, and low energy consumption, this technology possesses unprecedented potential for the evolution of display industry. Despite these advances, it struggles with the formidable technical challenge of mass transfer and still necessitates different substrates to manufacture LEDs of varying wavelengths.^[^ ^9^ ^]^ As Samsung has proposed, the OLED industry is also progressing toward blue OLEDs equipped with down‐converting QDs. Consequently, the industry's preferred solution is integrating the backlight unit with nanomaterial color‐conversion layer.^[^ ^10^ ^]^ Quantum dots and related semiconductor nanoparticles are being extensively investigated for their potential applications in future display technologies.^[^ ^3^, ^11^ ^]^ These materials can achieve narrowband photoluminescence through the quantum confinement effect. Furthermore, the luminescence color can be precisely modulated by controlling the size/composition of the quantum dots, thereby facilitating high color purity and broad color gamut.^[^ ^12^ ^]^ However, the use of these nanomaterials as PLCF is a non‐trivial task.

**Figure 1:**
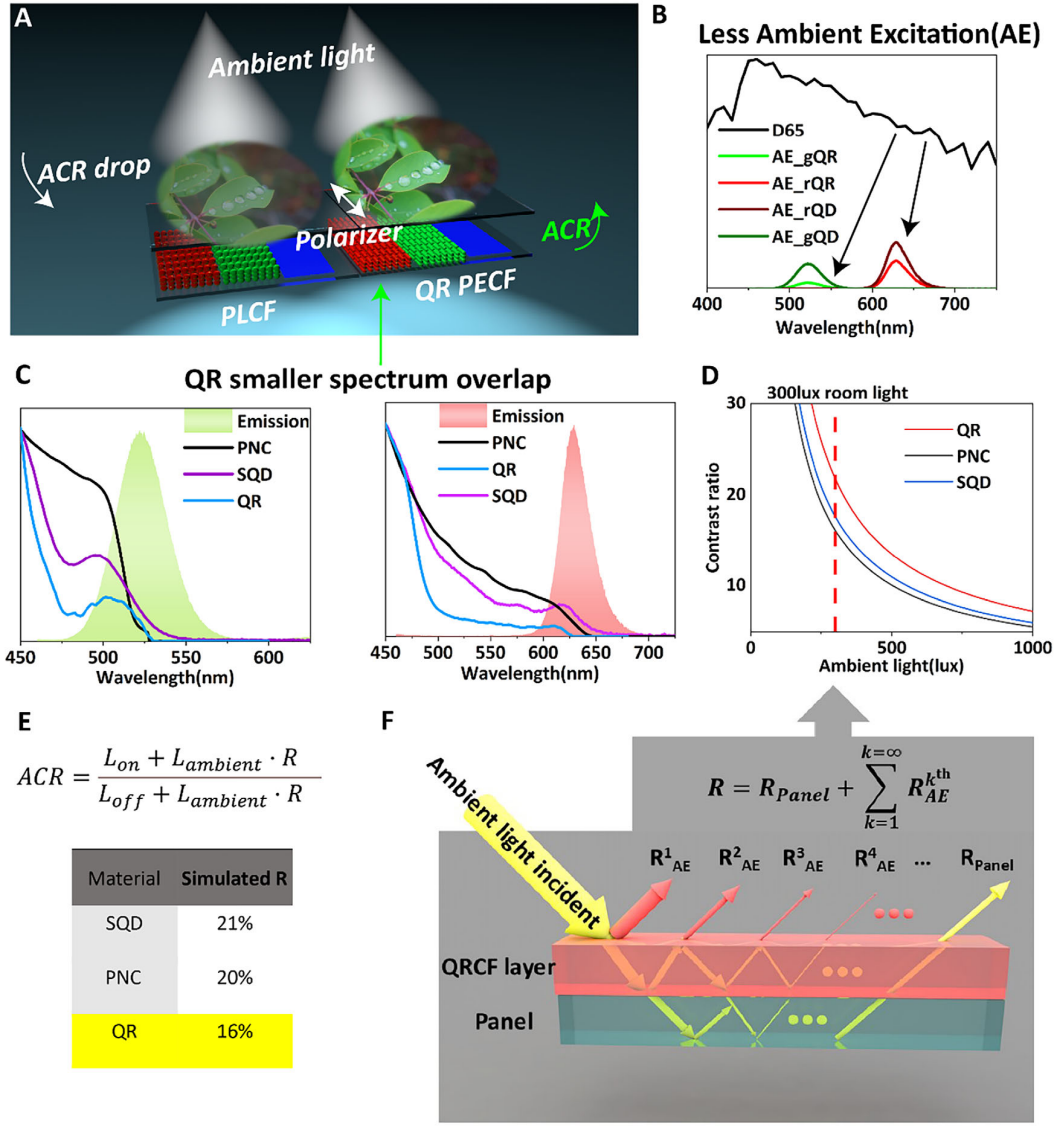
A) Schematic illustration of the problem of environmental light effect induced ACR drop and the illustration of the PECF and PLCF comparison; B) The simulation result difference for ambient light excitation of QDCF and QRCF; C) Absorption (normalized at 450 nm) and emission spectra relative overlap for different materials; D) Simulated ACR of the display image and E,F) The simulation model of ACR calculation and calculation result of ambient light excitation fraction.

The ambient contrast ratio (ACR) is an important characteristic for displays, particularly for portable devices. The ACR refers to the degree of contrast ratio between the light and dark states of a display under a given level of ambient illumination, which can be defined by the formula:

(1) $$ACR=\frac{L_{on}+L_{ambient}\cdot R}{L_{off}+L_{ambient}\cdot R}$$

here, *R* is the reflectance, *L_on_* is display brightness and *L_ambient_* is the ambient light brightness. It is clear from Equation (1) that the ACR of a display depends not only on its ability to emit light in the bright state, but also on its ability to stay dark in the dark state. Thus, the ACR drops significantly in the ambient lighting illumination conditions. Currently, there are two primary approaches to suppress the ambient light effect. One method involves reducing surface reflection, such as by applying anti‐reflective coatings on the ambient side.^[^ ^13^ ^]^ Alternatively, the use of circular polarizers can help eliminate the influence of external light.^[^ ^14^ ^]^ For fluorescent materials, such as semiconductor quantum dots, the ACR is a critical problem. Due to large total absorption of QDs in the visible range, the ambient illumination can undesirably excite these materials, causing poor display ACR (**Figure** **1**). These ambient excitations (AE) are generally defined as the emission of light from the photo‐luminescent material pumped by an external light source. The process of AE is quite complex and involves multiple reflections and re‐absorption processes, and multiple light paths need to be considered in complex device structures. Thus, rationally better CF strategies for modern displays to minimize the ambient impact may include optimizing the photoluminescent materials with relatively sharper drop of absorption from blue to green/red, compared to QDs (see Figure 1c), or designing a bottom‐emitting devices and anti‐reflective structure.

The second approach focuses on increasing the light output in the bright state. This requires careful design of the optical structure of the device to minimize light loss within the device, or the addition of external light extraction structures to enhance brightness.^[^ ^15^ ^]^ Some studies have also explored the introduction of scattering phenomena to improve ACR.^[^ ^16^ ^]^ However, these strategies necessitate a trade‐off between anti‐reflective performance and light extraction efficiency (LEE) to achieve an optimal balance of ACR and light output.

For practical applications, manufacturing QDCF in a pixelated and patterned manner also presents a challenge. It requires precise positioning and fixation of QDs while preserving their optical properties, such as luminescent efficiency and color purity. Moreover, to scale up the fabrication of these CFs, one needs to identify a swift, cost‐effective, and repeatable patterning method. Various fabrication methods, such as photolithography,^[^ ^17^ ^]^ inkjet printing,^[^ ^2^, ^3^, ^18^ ^]^ mold filling,^[^ ^19^ ^]^ thermoplastic molding,^[^ ^20^ ^]^ in situ synthesis,^[^ ^21^ ^]^ electrohydrodynamic (EHD) printing,^[^ ^22^ ^]^ and 3D printing,^[^ ^23^ ^]^ have been reported, but they are not mature enough.

In recent years, QR has garnered substantial attention from the research community. The QRs, with their elongated shape, larger UV‐blue absorption cross‐section,^[^ ^24^ ^]^ faster radiative decay rate,^[^ ^25^ ^]^ and advanced optical properties,^[^ ^26^ ^]^ represent greater advantages for the development of CF technology.^[^ ^27^ ^]^ Additionally, QRs offer higher LEE for the in‐plane aligned QRs than that for QDs^[^ ^28^ ^]^ and simultaneously have relatively less reabsorption than QDs, thereby reducing the emission losses. Thus, QRs possess a great potential for the efficient color converting layers. The latter also introduces a potential method for enhancing the ACR of the display in various ambient conditions (Figure 1b–e).^[^ ^29^ ^]^ The QRs, due to the dipolar emission and dielectric confinement effects, can emit linearly polarized light.^[^ ^26^, ^30^ ^]^ When QRs are unidirectionally aligned in the color conversion layer, macroscopic polarized emission can be achieved and optical efficiency of display can be improved due to increase in transmittance of the second polarizer, compared to QD CFs (**Table** **1**). These polarized emission color filters (PECF) have the potential to achieve high color saturation, wide viewing angle, high efficiency, and naturally high ACR and can be beneficial for the development of next‐generation display technologies. The PECF proposed in this work has two potential advantages over the QDPLCF: higher ambient contrast and higher energy efficiency, low internal device loss in case of in‐cell PLCF configuration, see Table 1, which are important attributes in future new displays. The main difficulty at present is how to increase the film thickness to achieve a practically important optical density (> 1) while ensuring high QRs alignment.

**Table 1:** Optical efficiency of LCD with QD and QR CFs.

QD‐LCD			QR‐LCD		
Components	Efficiency [%]	Brightness [%]	Components	Efficiency [%]	Brightness [%]
Backlight	100	100	Backlight	100	100
Diffuser	75	75	Diffuser	75	75
Polarizer	45	33.75	Polarizer	45	33.75
Glass	95	32.06	Glass	95	32.06
TFT	65	20.84	TFT	65	20.84
LC	85	17.71	LC	85	17.71
QDCF	65	11.51	PECF	65	11.51
Analyzer	45	5.18	Analyzer	70	8.05
Glass	95	4.92	Glass	95	7.66
Image		4.92	Image		7.66

Here, we studied a photolithography approach using QRs mixture with liquid crystal polymer (LCP) for fabricating pixelated PECF. Typically, two approaches had been studied in the past: the first involves mixing the PL material with a photoresist, and second, applying a patterned photoresist onto the PL material layer, followed by continuous illumination and high‐temperature baking for development and etching.^[^ ^31^ ^]^ This can inflict damage on nanomaterials with large surface area to volume ratios, thereby affecting their performance. Over the years, several improvements have been made, such as the addition of an indirect protective layer: depositing the pixel pattern onto a sacrificial layer, then transferring the pattern from the sacrificial layer to the QD layer, effectively reducing impurity residues and material PL loss.^[^ ^17a^ ^]^ The other approach employs photo responsive ligands to modify QDs or photoactive agents to directly pattern the QD layer.^[^ ^32^ ^]^ It typically involves combining inorganic nanomaterials and organic solvents. Although this method may partially damage QD color conversion layer, it greatly retains the inherent PL characteristics of the QDs. In this work, by using photoreactive LC monomer, we were able to ensure the luminescent properties and alignment of the QRs during the fabrication process. Thus, the proposed approach to achieve pixelated, polarized, QR‐based PLCFs presents immense potential for efficient displays with high ACR. **Table** **2** compares different technologies used in nanomaterial‐based color conversion films.

**Table 2:** Comparison of published works on nanomaterial‐based color conversion films.

DOP	ACR (room light)	Pixels	Full color	PLQY [%]	Material	Alignment method	Ref.
0.35	n/a	Y	N	77%	QR(R)	Langmuir‐Blodgett (LB) technique	[33]
0.89	n/a	N	N	40%	QR(R)	Capillary forces	[34]
0.48	n/a	N	N	NA	QR(R)	Electric‐field‐aided alignment	[35]
0.6	n/a	N	N	NA	QR(R)	Electrospinning	[36]
0.56	n/a	N	N	NA	QR(R)	Mechanical rubbing	[37]
0.62	n/a	Y	N	84%	QR(R)	Photoalignment	[38]
0.6	n/a	N	Y	93%	QR(RG)	Mechanical stretching	[39]
0.63	n/a	N	Y	75%	QR(RG)	Photoalignment	[40]
n/a	58.1:1	Y	Y	n/a	QD(RG)	Photolithography	[41]
n/a	189:1	Y	Y	75%	QD(G)QR(R)	Inkjet‐printing	[2]
0.65	263:1	Y	Y	82–98%	QR(RG)	Photoalignment	This work

In this study, we utilize QR's linearly polarized emission property to develop PECF, for the first time. First, we revisited and improved the method to synthesize green emitting QRs with good optical properties followed by the fabrication of the PECF using photo‐alignment. The fabrication process includes ligand exchange for the QRs, mixing them with liquid crystal monomer (LCM) followed by alignment, patterning, and curing to form pixelated CF films. The quality of alignment and fixation of the PECF demands high compatibility between the LCM and QRs. By leveraging T‐ligand QRs, we achieved superior dispersion of QRs, at higher concentrations, in the LCM that also produced a uniform intact film. The fabricated films are robust enough against multistep alignment and photolithography process. We studied two techniques: photolithography, and inkjet printing, to pixelate the color conversion layer for a full‐color conversion. The single pixel of the PECF exhibits a degree of polarization (DOP) ≈0.66, for red and green CFs. The QR‐based PECFs offer high ACR of 263:1 at an ambient luminance of 200 lux. The findings in the work highlight the potential of PECFs as a promising alternative to traditional CFs, paving the way for the realization of high‐performance displays with enhanced efficiency, thereby highlighting the promising outlook of this technology in the realm of advanced display applications.

## 2 Experimental Section

### 2.1 Synthesis of Green (≈520 nm) Emitting QRs

CdSe/CdS QRs (λ_max_ = 578 nm, FWHM = 31 nm) were synthesized according to the seeded growth approach.^[^ ^42^ ^]^ To the prepared CdSe/CdS QR reaction mixture (4.0 mL, ≈0.42 mmole of Cd), without an isolation and purification of the QRs, a mixture of 140 µL of silver nitrate solution in trialkylphosphine oxide (11 mm, contains also ≈30 mm of oleic acid) and 5.7 mL of zinc oleate solution in 1‐octadecene (0.32 m) was added dropwise during 2 h at 330 °C. The reaction mixture was stirred at 330 °C for 1 h more and then cooled down to room temperature to stop the reaction. The resulting QRs were isolated by deposition with isopropanol and centrifugation and washed three times by reprecipitation using toluene‐ethanol solvents combination. The final product was dissolved in chlorobenzene in concentration 25 g L⁻^1^. Red emitting rod‐in‐rod QRs were prepared according to the procedure from ref. [28b].

### 2.2 Ligand Exchange Procedure

**Figure 2:**
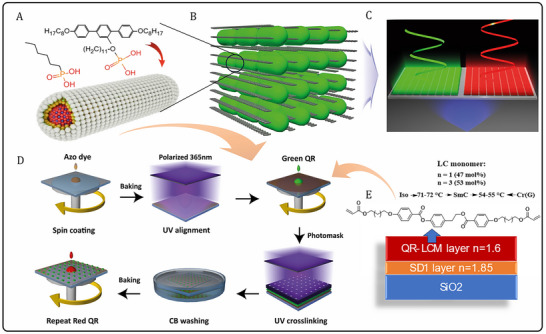
Schematic illustrations of A) Quantum rod (QR) with T‐shape and HPA ligand combination; B) The photoaligned QRs; C) PLCFs with polarized emission; D) PLCF fabrication process; E) CF layers structure and LCM molecular composition.

The synthesis of T‐shape ligand (T‐ligand, **Figure** **2**A; Figure S9, Supporting Information) is described in ref. [40]. For the ligand exchange, the QRs solution (≈40 mg of QRs) was evaporated under nitrogen purging, and 3.0 mL of trioctylphosphine was added to dissolve the QRs. A total of 150 nmol of T‐ligand (TL) and hexylphosphonic acid (HPA) were added to each sample at a molar ratio of TL:HPA = 1:9. The mixture was thoroughly degassed by vacuuming for 1 h at room temperature, followed by five cycles of vacuuming and nitrogen filling. The solution was then heated to 160 °C and stirred for 2 h. Afterward, the reaction mixture was cooled to room temperature and diluted with ≈2 mL of toluene. The ligand‐exchanged nanorods were precipitated using ≈3 mL of ethanol, and the centrifuged precipitate was washed twice by dissolving in toluene and reprecipitating with ethanol. The purified ligand‐exchanged QRs were dissolved in chlorobenzene, centrifuged, and filtered through a 0.2‐micron PTFE microfilter to produce a final QR solution with a concentration of 25 g L⁻^1^. These solutions were further diluted with 1,2‐dichlorobenzene as needed for QR ink preparation, as described below.

### 2.3 Ink Preparation

The inkjet printing used a mixture of QRs and LCM as ink, with chlorobenzene and 1,2‐dichlorobenzenes as solvents (mixed 1:1 by volume). The LCM was a mixture of two homologues of 4‐(2‐((4‐(3‐(acryloyloxy)alkoxy)benzoyl)oxy)ethyl)phenyl 4‐(3‐(acryloyloxy)alkoxy)benzoate possessing smectic C mesophase in the individual form (Figure 2E). The QR solution was prepared at a concentration of 20 mg mL⁻^1^ in chlorobenzene (CB). In the ink, the mass fraction of QR is 0.5%, while the mass fraction of LCM is 10%. To achieve a homogeneous mixture, the components were combined and subjected to 15 min of ultrasound treatment. These specific compositions and concentrations were optimized to ensure the desired performance and functionality of the ink for the specific application. Photolithography used the same blend of QR and LCM, but the solvent was individual chlorobenzene.

### 2.4 SD1 Film Coating and Photoalignment

A sulfonic azo dye molecule (provided by Dainippon Ink and Chemicals Ltd., Japan (DIC)) was used as the light‐orientation layer by first dissolving SD1 in dimethylformamide (DMF) to obtain a solution with 1 wt.% concentration. The solution was then drop‐coated onto a glass substrate by spin‐coating, and then the substrate was placed on a heating table at 100 °C for 10 min to evaporate the excess solvent. Finally, the substrates were exposed to polarized UV LED (370 nm; 5.63 mW cm⁻^2^) irradiation for 200 s at room temperature and humidity ≈60% to obtain SD1 photoaligned film (Figure 2D).

### 2.5 Mask Fabrication

The mask plate used for lithography was a contact mask plate fabricated by chromium plating on a transparent glass substrate, and the pattern consisted of a square matrix with a pitch of 500 microns and a side length of 300 microns.

### 2.6 Lithography Pixelated CF Fabrication

The red QR‐LCM blend solution was spin‐coated on the substrate, and the liquid crystal grid was cured using polarized UV light for 300 s; while curing, the film was exposed using a pixelated metal mask, and the exposed substrate was soaked in a chlorobenzene solution for 5 h (Figure 2D). The soaked substrate was cleaned using a chlorobenzene solution on a spin‐coating device. Then the same substrate was spin‐coated with green QR‐LCM solution, and the same steps were repeated to control the pattern of the final CF pixels by controlling the relative position of the masks.

### 2.7 Material Characterization

Transmission electron microscope (TEM) images were observed by the JEM 100CXII (JEOL) instrument. QRs PLQY, absorption, excitation anisotropy and PL spectra were measured by FS5 spectrofluorometer (Edinburg instruments). The structure and composition of gradient alloy QRs were analyzed by elemental mapping using energy dispersive X‐ray spectroscopy (EDS) combined with a TEM operating in scanning transmission electron microscope (STEM) mode. The distribution of elements along the diameter of the QRs was plotted, and they were fitted with a normal distribution curve. A comparison of the distributions of zinc, cadmium, and sulfur was done by comparison of *σ* parameter from the normal distribution equation (variance) reflecting the curvature of the distribution. Silver‐catalyzed QRs showed lower variance (steeper transition) for Cd and higher variance (shallower transition) for zinc at the scanning along the QR diameter. Conversely, the copper‐doped QRs exhibited higher cadmium element concentration near the core.

### 2.8 Optical Setup and DOP Measurement

Fluorescence photographs of the samples were observed and taken using a fluorescence microscope (Murzider MSD‐S280). To accurately measure the DOP of a single pixel filter, a laser source was used with a wavelength of 405 nm, a lens to focus the spot into a pixel under a microscope, and an Ocean Optics USB4000 spectrometer was used to record the emitted light (Figure 5D). A line polarizer was placed before the detector, and the line polarization was rotated to obtain the luminous intensity at different pinch angles, and the value of DOP was obtained by calculation. DOP is defined as:

(2) $$DOP=\frac{I_{max}-I_{min}}{I_{max}+I_{min}}\;$$

where *I_max_* and *I_min_* are the maximum and minimum values measured by the data captured during the analyzer rotation process.

### 2.9 Device Fabrication

A blue (450 nm) LED backlight was used as the excitation light source to manufacture the full‐color display demo. A PLCF pattern that is 1.5 cm long, 300 µm wide, with a pixel pitch of 100 µm, was printed. ITO electrodes with the same resolution were also printed to manufacture the liquid crystal cell.

For the ACR test device, the same process was followed to manufacture a 5 × 5 mm^2^ pattern and printed ITO glass of the same area to manufacture the liquid crystal cell.

### 2.10 Device Measurement

The device was placed on a stable optical bench, and the spectroradiometer (Konica Minolta CS‐2000) was pointed at the display; the measurement area was aligned on the pixels, the pixels were switched on and off by controlling the drive of the LCD cell, and the luminous flux was measured.

### 2.11 Simulation

The simulation of light distribution and device ACR was done using a combination of Matlab and Ansys Lumerical software. For the ACR simulation of the CF, the model is shown in Figure 1F. The emission spectrum data and absorption spectrum data are from the actual fluorescence spectra measurements using spectrometer (FS5 Edinburgh Instruments). Ansys Lumerical was used for Finite‐Difference Time‐Domain (FDTD) simulation. The reflectivity settings and model structure of the material are shown in Figures SI‐4 and 5 (Supporting Information).

## 3 Results

### 3.1 Material Synthesis

There are two key parameters we consider for improving PLCF performance in displays: ambient light excitation and polarized PL emission. Considering the requirement for the optical density (> 1)^[^ ^43^ ^]^ at the wavelength of excitation (*λ_ex_*), the first parameter primarily depends on the difference in material absorption at the *λ_ex_* (*µ_450_*, based on typical commercial blue LEDs) and in the visible wavelength range (*µ_vis_*). When normalized to 450 nm absorption, QRs show relatively lower absorption in the visible range, compared to commercially available QDs and perovskite nanocrystals (see Figure 1C and ref. [28b]). While the absorption properties of QDs can be enhanced through compositional modifications, such as increasing the relative amount of wider‐bandgap semiconductor (e.g., CdS, ZnSe) or synthesizing giant‐shell QDs, the PL emission of QDs still lacks polarization, which is important for improving LCD optical performance, particularly in in‐cell PLCF configuration. In this context, 1D semiconductor nanorods (QRs), nanowires (NWs) or 2D nanoplatelets (NPLs) are potential candidates, with QRs being the preferred choice due to their superior colloidal stability, easier large‐scale alignment, and patterning processability.^[^ ^27^, ^40^, ^44^ ^]^

**Figure 3:**
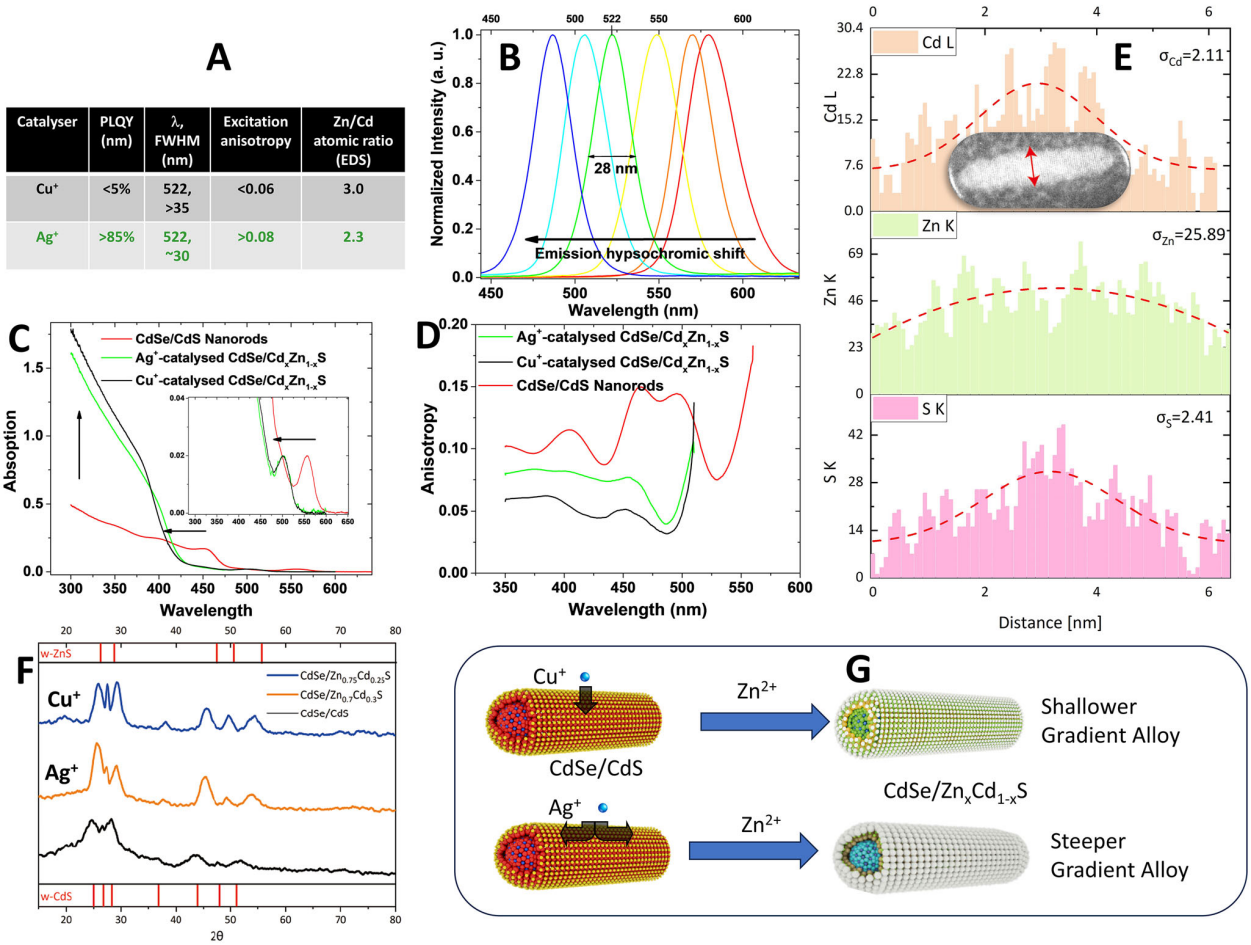
QR material characterization. A) Comparison of properties of CdSe/Zn_x_Cd_1‐x_S QRs prepared through catalysis with Cu^+^ and Ag^+^ properties; B) PL emission trend in synthesis of CdSe/Zn_x_Cd_1‐x_S QRs; C) Absorption and D) Excitation anisotropy trends in synthesis of green emitting CdSe/Zn_x_Cd_1‐x_S QRs through Cd^2+^ to Zn^2+^ cation exchange using Cu^+^ and Ag^+^ catalysis; E) Radial EDS elemental mapping results for single CdSe/Zn_x_Cd_1‐x_S QRs prepared using Ag^+^ catalyzer; F) Powder X‐ray analysis results for CdSe/Zn_x_Cd_1‐x_S QRs; G) Schematic of Cd^2+^ to Zn^2+^ cation exchange process using different catalyzers.

In this work, we utilized rod‐in‐rod CdSe/CdS/ZnS QRs, leveraging their higher stability due to the ZnS shell.^[^ ^45^ ^]^ These QRs were prepared following the previously proposed method.^[^ ^28b^ ^]^ Using rod‐shaped CdSe nanoparticle seeds enhances optical anisotropy, thereby increasing the polarization degree of the final QRs.^[^ ^46^ ^]^ For green emitting CdSe/Zn_x_Cd_1‐x_S QRs, we first conducted the seeded growth of dot‐in‐rod CdSe/CdS QRs followed by Cd to Zn exchange in the presence of Ag^+^ catalyst to facilitate the cation exchange process. Previously, Cu^+^ cation was shown to effectively catalyze the Cd to Zn exchange process in CdSe/CdS QDs, resulting in alloyed CdZnSe/CdZnS core/shell nanoparticles, though with a moderate quantum yield for the final product.^[^ ^47^ ^]^ It is known that even a small amount of Cu impurities in QDs drastically reduces their quantum efficiency.^[^ ^48^ ^]^ We systematically studied the role of different soft metal cations in the Cd^2+^ to Zn^2+^ cation exchange process. We found that by using Ag^+^ catalysts, one can achieve the final QRs with great optical properties in terms of PLQY, FWHM and anisotropy. Furthermore, we also studied various protocols for adding catalysts and Zn‐precursor, including sequential Ag^+^ and Zn‐precursor injections, both forward and backward sequences, slow addition of either catalyzer or Zn‐source, and mixing of these two in a single injection solution. Finally, we elaborated an optimized protocol in which a catalytic amount of inorganic silver salt (silver acetate or nitrate) is mixed with Zn precursor (solution of zinc oleate in 1‐octadecene). This mixture is slowly added to the preformed CdSe/CdS QR in the reaction mixture one‐pot, resulting in a smooth blue shift of PL peak, narrow emission bandwidth (**Figure** **3**B) and high PLQY, particularly for the green emitting QRs. We found that no subsequent removal of residual Ag^+^ is required as the PLQY of the resulting QRs is already approaching unity for green‐emitting samples.

It is noteworthy that although the evolution of the PL spectra exhibited similar trends for Cu^+^ and Ag^+^ catalyzed cation exchange processes, there was a clear distinction in other properties of the resulting products (Figure 3). For comparison, we synthesized green‐emitting (522 nm) CdSe/Zn_x_Cd_1‐x_S QRs using two different catalysts starting from the same reaction mixture of CdSe/CdS QRs. Both the UV–vis absorption spectra (Figure 3C) and EDS elemental analysis revealed a higher Cd‐to‐Zn conversion in the Cu^+^‐catalyzed process with a Zn‐to‐Cd ratio of 3, compared to Zn/Cd = 2.3 in the case of the Ag^+^ catalyst, despite the same emission wavelength for these two samples. X‐Ray crystallographic analysis revealed a shift of the characteristic reflections from *w*‐CdS to *w*‐ZnS angles for both samples (Figure 3F). We studied the elemental distribution within the CdSe/Zn_x_Cd_1‐x_S QRs through a combination of STEM and EDS elemental mapping. Comparing the Cd and Zn distribution in the Ag^+^‐catalyzed QRs clearly indicates a gradient alloy structure (see Figure 3E) with a sharp transition from CdS to ZnS from the center to the shell of the rod. In contrast, for Cu^+^‐catalyzed QRs, a more homogeneous alloy is formed with a shallower CdS→ZnS transition (Figure S8, Supporting Information), which may explain a significant difference in the optical properties of the QRs (PLQY and anisotropy, Figure 3D) despite the same emission wavelength. The replacement of Cd^2+^ by a soft catalyst cation in the CdS crystal lattice is the driving force behind the entire process of exchange of Cd to Zn. In terms of HSAB (Hard Soft Acid Base) theory, Ag^+^ and Cu^+^ have very close hardness indices,^[^ ^49^ ^]^ being both soft acids in contrast to Cd^2+^ and Zn^2+^, which are both much more harder bases. Thus, thermodynamically the reaction is driven by stronger binding of catalyst ions with a very soft base – sulfide (S^2−^) anion within the CdS crystal lattice. We assume that the reason for the different final distribution of Cd and Zn within the resulting Zn_x_Cd_1‐x_S shell is the different ion mobility of Cu+ and Ag+ within the CdS crystal lattice. CdS shell has wurtzite crystal which has a unique crystallographic *C*‐axis. It was experimentally shown that mobilities of Cu^+^ and Ag^+^ ions are not equal in parallel and perpendicular to *C*‐axis directions in CdS and CdZnS wurtzite crystals.^[^ ^50^ ^]^ Thus, mobility of Cu^+^ is more than two orders of magnitude higher in direction perpendicular to *C*‐axis, while Ag^+^ shows several times higher mobility parallel to the *C*‐axis. As a result, one can expect more substitutional defects created by Cu^+^ ions toward the inner core of QRs, whereas in case of Ag^+^, these defects should be more distributed at the surface of QR slowly moving to the core during the cation exchange process. These Cu_Cd_ and Ag_Cd_ centers are further substituted by Zn^2+^ ions, which are in large excess in the reaction mixture, thereby leading to more homogeneous, in case of Cu^+^, and more gradient‐like, in case of Ag^+^, Zn_x_Cd_1‐x_S structures (Figure 3G). We believe that the gradient alloyed shell structure in the case of Ag^+^ catalyzed green QRs and higher tolerance of QR optical properties to small amounts of the residual silver are responsible for higher PLQY of these rods, due to less crystal lattice mismatch and less amount of the residual crystal defects.

### 3.2 Film Uniformity Optimization

**Figure 4:**
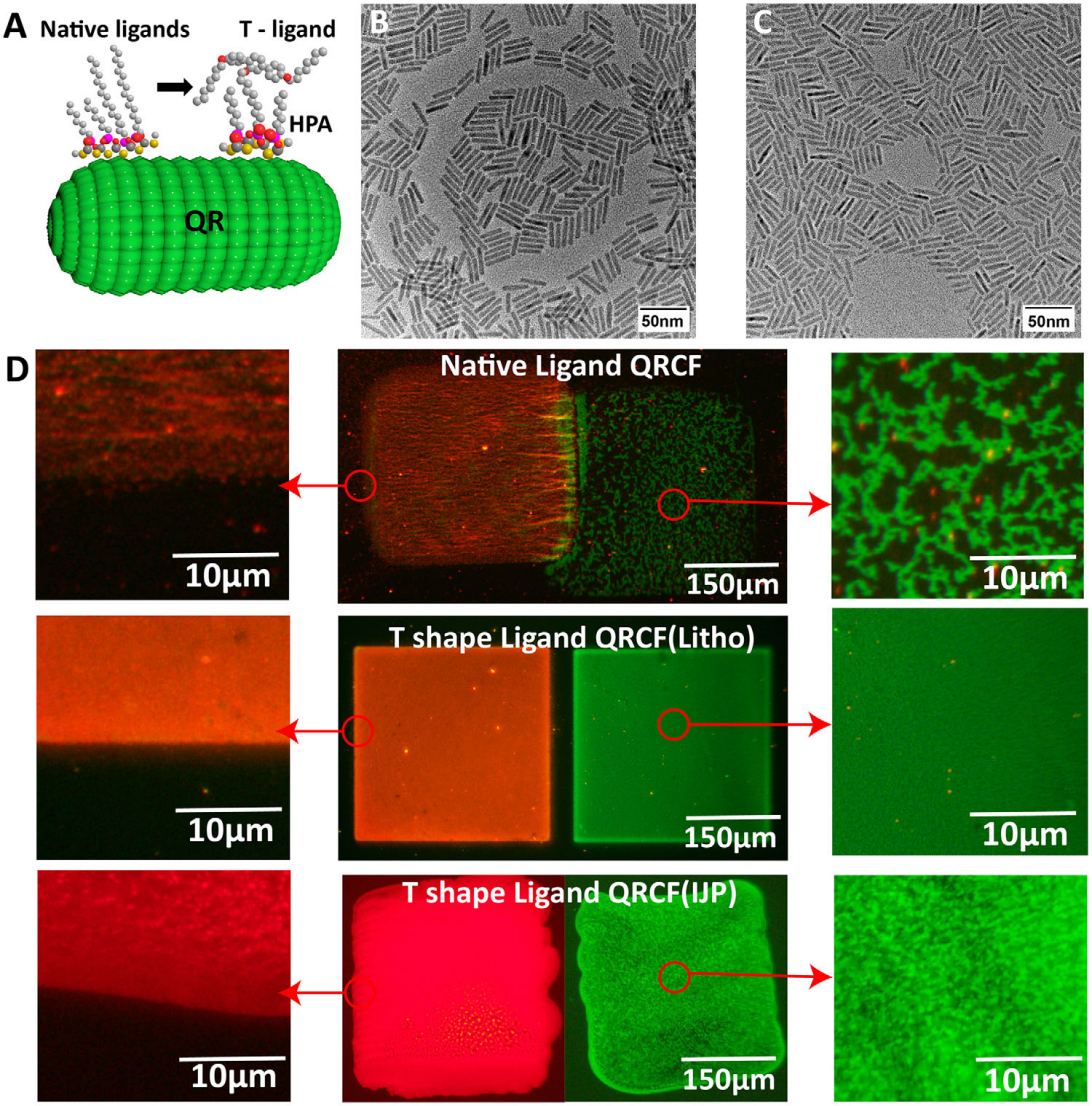
A) QR for T‐shaped ligand exchange. TEM image for B) green QR and C) red QR. D) PLCF PL microscope image comparison of native and T‐ shape ligands.

Film uniformity of CF is a challenging task during the manufacturing process and is primarily governed by the concentration and solvation properties of fluorescent materials in a given solvent. For QRs in complex solvent systems, such as with the addition of LCM, the colloidal stability significantly decreases,^[^ ^51^ ^]^ and the quality of the fabricated film suffers from aggregation and fluorescence quenching problems. Further, the colloidal stability of nanoparticles with native aliphatic ligands is low in highly ordered (LC) medium.^[^ ^52^ ^]^ Specifically for QRs covered with native aliphatic ligands (alkylphosphonic acids mixture), a significant degree of aggregation has been reported within the LCM/LCP films.^[^ ^53^ ^]^ This is because the interaction between the native (aliphatic) ligands and LCM is weak as it is limited to only dispersion (London) intermolecular forces. Thus, surface ligand modification is required to solve the above mentioned problems. The use of vertically attached promesogenic (LC‐like) ligands can improve film homogeneity due to significantly stronger LCM‐QR interaction as it is driven by aromatic π‐π interaction, though the alignment improvement is limited to a very low concentration.^[^ ^53^ ^]^ To realize the effective alignment of QRs in high‐concentration films, a special T‐shape side‐anchoring promesogenic ligand was applied in combination with a short co‐ligand (hexylphosphonic acid) (Figure 2A and **Figure** **4**A).^[^ ^40^ ^]^ The ligand comprises a terphenyl derivative as the main body, capable for π‐π interaction with a LCP, and a phosphonate group on the lateral chain that can anchor onto the surface of QRs. Thus, in contrast to vertically attached ligands,^[^ ^53^ ^]^ the terphenyl rings in T‐ligand are arranged parallel to the long axis of the QR, which is the key to realizing their efficient alignment at high concentrations.^[^ ^40^ ^]^ The short‐chain alkylphosphonic acid is used as a co‐ligand to fix the optimal stereo conformation of T‐shape ligands, preventing the terphenyl ring from being tilted perpendicularly to the long axis of the QR through the steric repulsion effect. In addition, using an optimized TL/HPA molar ratio (1:7–1:10) improves the solubility of QRs in both conventional solvents and within LCM‐medium and minimizes the perturbation of the resulting LCM/LCP molecules alignment. After the azo dye layer is photoaligned, the LCM orientation can be transferred to the QRs through the LCM‐ligand interaction and the mixture is polymerized. By changing the light polarization direction for the alignment of the azo dye, the QRs can be patterned and oriented. The method enables a straightforward control of the QR alignment in the microscale and realizes the effective large‐scale alignment of the QRs.^[^ ^26^, ^27^, ^40^, ^54^ ^]^

It can be observed (Figure 4D) that the T‐ligand modified QRCFs show high film uniformity, whereas, QRs with the initial (native) ligand are completely aggregated with no PL emission observed between the micron‐size conglomerates. Non‐uniform CFs can lead to serious color crosstalk and light leakage affecting the color performance of the display device. Simulation of the system using FDTD method revealed that nonuniform CFs has a relatively higher light coupling efficiency; but, due to the randomly distributed nanoparticle aggregates therein, part of the light will be scattered and lose the polarization nature with additional light losses in the device overall (Figures S4 and S5, Supporting Information). Ideally, a completely uniform film layer could achieve a transmittance of ≈80%. As the film uniformity increases, scattering losses first increase, reaching a maximum at a certain specific value and then gradually decrease. It implies that a non‐uniform film can lead to a light loss of ≈0.5% to 8%. We confirmed the simulation results by measuring with spectroradiometer the light output of two types of QRCFs with the same manufacturing process and nearly identical thin‐film quantum yields. The experimental results showed that the T‐ligand CFs had a 0.5% to 2.3% increase in transmittance relative to the native ligand CFs, which is consistent with our simulation results.

### 3.3 Polarized Emitting Color Filters Design and Characterization

**Figure 5:**
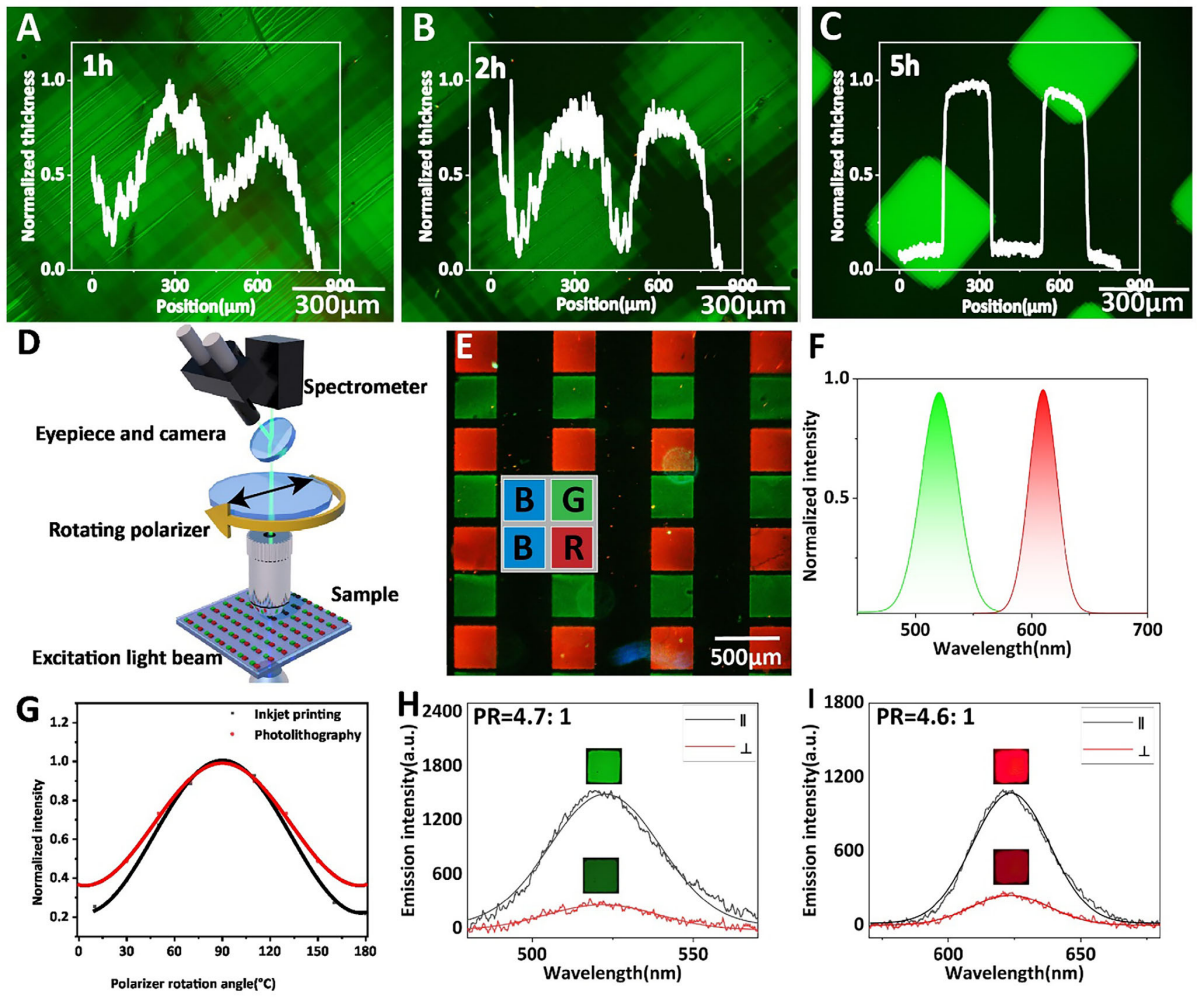
The FM image and diagonal brightness grayscale distribution curve of A) 1 h, B) 2 h, C) 5 h rinsed PLCF film; D) Schematic illustrations of the DOP measurement of PLCF pixel; E) The FM image of GB PLCF array; F) The PL spectrum of green and red PLCF; G) PL intensity change of the PLCF with varying polarizer angles of different fabrication ways; Parallel and perpendicular polarizer state PL spectrum for inkjet printed H) green; and I) red PECF.

Compared with the electric field‐driven QR alignment method, which requires a very high electric field and a specific design of electrodes,^[^ ^35^ ^]^ the light‐driven QR alignment adopted in this study is relatively easier. The photoalignment process includes a coating of azo dye layer followed by irradiation with polarized light. The easy axis of the alignment aligns perpendicular to the polarization azimuth of the irradiating light.^[^ ^26^, ^27^, ^40^ ^]^ Furthermore, by changing the polarization azimuth of the irradiating light, one can alter the easy axis of the photoalignment and the QR's alignment direction, enabling more flexible local patterning. Unlike the mechanical stretching method,^[^ ^39^ ^]^ which may cause mechanical damage to the material, the present photoalignment method can maintain the morphology of the film, and precisely define the alignment direction of the oriented zones/pixels.^[^ ^40^, ^55^ ^]^ The curing of LCM after photoalignment results in a stable polymerized structure, ensuring long‐lasting performance, which is also essential for the subsequent layer depositions. To avoid dissolution of the bottom alignment layer in the subsequent CF fabrication process, we used polar bis‐azo dye (SD1) for the photoalignment process, which cannot be dissolved by non‐polar solvents used for QR‐LCM ink. The QR‐LCM hybrid layer was spin coated on top of the pre‐aligned SD1 film; thereafter, the substrate was exposed to UV light through a shadow mask to polymerize the LCM and fix the alignment direction and pixels positions, similar to positive photoresist in photolithography process (Figure 2D). The non‐exposed film areas on the substrate were then washed out by immersion in chlorobenzene. Afterward, the process was repeated for green‐emitting PLCFs array. For the cleaning process, we tested different time durations for 1 h, 2 h, and 5 h, respectively, in chlorobenzene. We observed a correlation between these temporal variations and the subsequent integrity of the resultant pixelated pattern. As the immersion interval extends, the unexposed regions of LCM, loaded with QRs, gradually dissolve into the chlorobenzene (**Figure** **5**A–C). The regions proximate to the exposure area present greater resistance to this dissolution process due to the higher cross‐linking density of the polymer network caused by the photopolymerization reaction. Nevertheless, following a prolonged immersion period of ≈5 h, all undisturbed regions are effectively eliminated. Consequently, as illustrated in Figure 5E, a precise and homogenous pixelated pattern is achieved. Extending the cleaning duration beyond 5 h risks compromising the integrity of the original thin film, reducing its thickness, and ultimately degrading its optical performance (Figure S2, Supporting Information). With optimized washing time we achieved contours with remarkably straight edges and uniform emission (Figure 5C,E,F). The DOP of the pixels was measured to be 0.52 and 0.51 for green and red PECF, respectively. By manipulating the orientation of the polarizer (Figure 5D), we observed the correlation between luminous intensity and the polarizer angles following the Malus's law (Figure 5G). The near‐identical value of the DOP for red and green pixels confirms a stable and intact SD1 alignment layer and LCP film during the CF layer deposition and washing in chlorobenzene.

**Figure 6:**
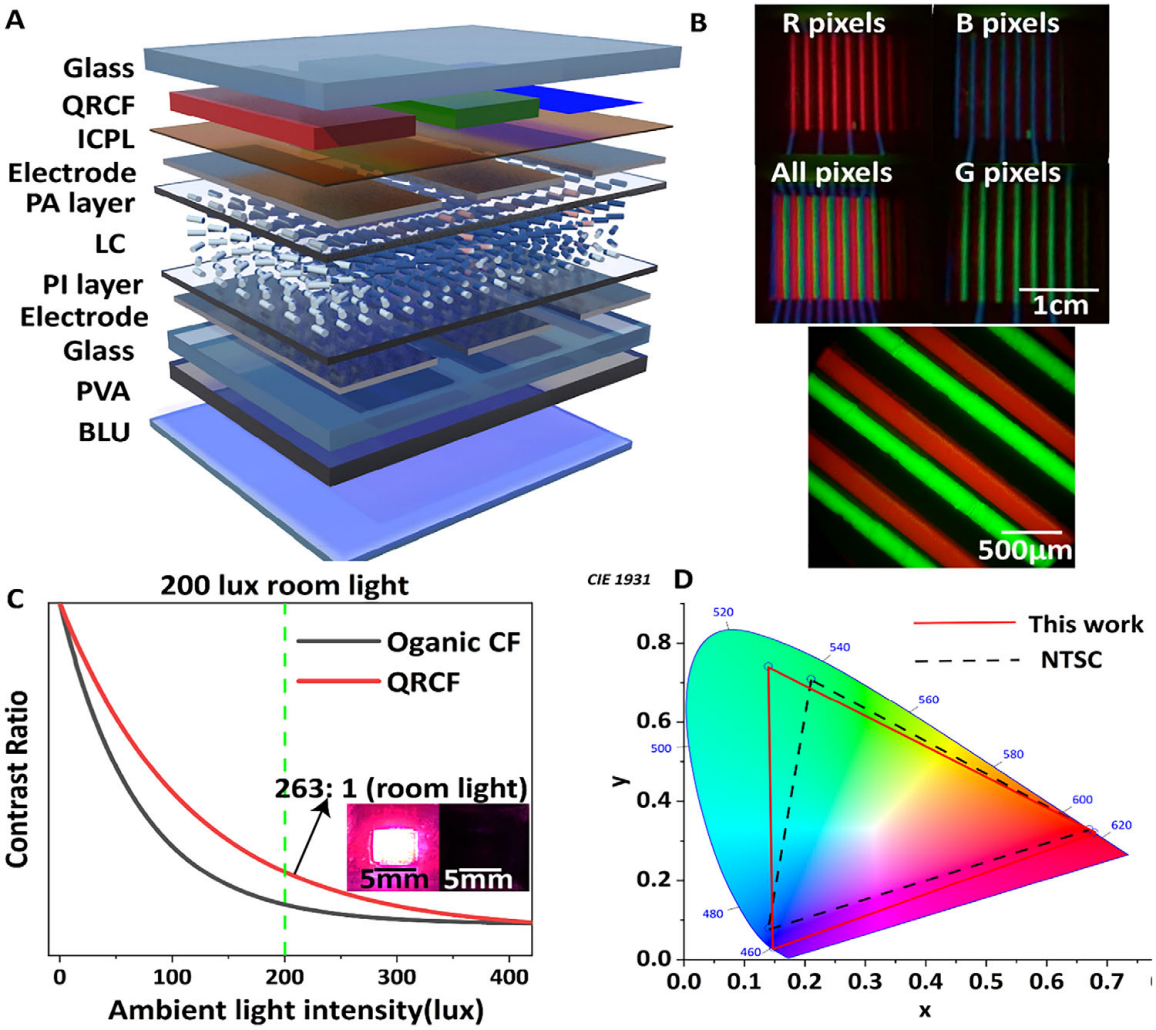
A) Schematic illustration of top emitting PECF full color LCD device; B) Different pixels on/off state and micro image of the fabricated PECF displays; C) ACR comparison of QRCF and organic dye CF; D) The color gamut for device of CIE1931 color space.

The spin‐coating photolithography technique provides a great flexibility in utilization of an extensive range of solvents and materials. However, it suffers from several drawbacks. Issues such as elevated material waste and challenges in achieving high uniformity have imposed limitations on its adoption in CF manufacturing, especially for a bigger display size. Thus, other coating techniques, such as inkjet printing (IJP), are a more viable alternative and should be studied for the proposed coating, polymerization, and washing processes. IJP permits the direct application of ink onto a transparent substrate, facilitating a swift and cost‐effective manufacturing process. Moreover, it allows for meticulous control over the size and position of the ink droplets, thereby enabling precision patterning with high uniformity and material utilization efficiency. For QR‐LCM mixture, 1:1 chlorobenzene/1,2‐dichlorobenzene (CB/DCB) solvent mixture enables high colloidal stability of QRs with no visible signs of QRs/LCM phase separation.^[^ ^51^ ^]^ In addition, the controlled viscosity and evaporation rate of this solvent mixture mitigate the coffee rings and satellite droplets problem, which can cause uncertainties in droplet placement, compromising the film and pixels uniformity. Using QD‐LCM‐CB/DCB ink, we printed red and green PECF pixels array and cured it with polarized UV light. The relative intensities of the emitted light are plotted as a function of the polarizer rotation angle (Figure 5G), and the DOP of the red and green QR PLCF, using the inkjet printing, increased to 0.64 and 0.65, respectively (Figure 5H,I). Finally, we deployed the fabricated PECF array on the LCD in a top‐emitting configuration (see **Figure** **6**A). We used a passively addressed LCD cell with patterned ITO and embedded a thin‐film polarizer in the LC cell.^[^ ^56^ ^]^ The polarized light‐emitting CFs effectively reduce the light loss in the LCD and show a remarkably high color gamut of 120% NTSC in CIE1931 (Figure 6D).

### 3.4 ACR Simulation and Measurement

ACR is an important parameter in the application of PLCF to display devices and it is defined as:

(3) $$ACR=\frac{L_{on}+L_{ambient}\cdot R}{L_{ambient}\cdot R}\;$$

here $R=R_{panel}+\sum_{k=1}^{k=\infty }R_{AE}^{k^{th}}$, where *R* is the reflectance, which mainly consists of two parts in case of photoluminescent CFs; first ($R_{AE}^{k}$) is the PL emission caused by ambient light excitation (AE) of the material, and second (*R_panel_*) is the reflection of the substrate and optical structure. *L_on_* is the display brightness and *L_ambient_* is the ambient light brightness.

In our simulation we investigated the interaction of incident light with a PLCF system, considering the absorption and emission spectrum of the material as well as their refractive indices. In addition, PLQY and LEE are considered to evaluate the conversion efficiency of the absorbed to emitted photons escaping the PLCF film. We also studied the secondary emission spectrum resulting from the absorption of reflected photons by the photoluminescent material. Finally, we derived the reflection coefficients from all aspects and calculated the corresponding ACR. We simulated quantum dots, luminescent organic dye, and QRs, and the results are summarized in Figure 1D. The simulated results show that the QRs‐based CFs offer higher ACR than the spherical particles, which can be attributed to their smaller overlap between the emission and absorption spectrum.

Considering this, we designed a display device based on the aforementioned polarized PLCF, incorporating twisted nematic liquid crystal pixels excited by a 450 nm blue LED backlight unit (BLU). Thereafter, we measured the pixel's luminance in both bright and dark states under various ambient light intensities (as depicted in Figure 6C). Moreover, we have compared the ACR for polarized QR PLCF with PLCF based on luminescent organic dye (DBP:DMP).^[^ ^4c^ ^]^ The results suggest that the ACR of the organic CF declines faster than the polarized QR PLCF when ambient light illumination increases. The ACR for QR‐PLCF shows more than twofold improvement at the ambient light intensity range of 100–300 lx, after which the contrast of both samples decreases significantly. Thus, less absorption of QRs in the visible range reduces AE, and thereby *R*, enhancing the ACR of the device. On the other hand, less reabsorption effect reduces the overall light losses in the “on” state (*L_on_*) increasing the display brightness. Although this study has demonstrated the advantages of the proposed technology in a pioneering way, there is still room for improvement. To further optimize the display quality, future work should focus on increasing the film thickness and conversion efficiency of the PECFs, enabling straightforward application in the future high‐performance displays.

## 4 Conclusion

In conclusion, this study emphasizes the significant potential of polarized light emitting CF technology as a promising alternative to traditional QD‐based displays. The careful material design, including QR synthesis and ligand shell on QR surfaces, as well as optimizing inkjet printing, photolithography, and photoalignment processes, allowed us to achieve a high DOP reaching 0.65 for accurately patterned QR CFs with pixel‐level precision. These CFs achieve full‐color representation, offering enhanced color saturation, ACR, and efficiency for display. The utilization of QRs in this context has notably addressed challenges related to color purity and light losses, thanks to their narrow‐band polarized emission and low reabsorption effect. This research opens up exciting possibilities for advancing display technologies with enhanced performance and visual quality.

## Conflict of Interest

The authors declare no conflict of interest.

## Supporting information



Supporting Information

## Data Availability

The data that support the findings of this study are available on request from the corresponding author. The data are not publicly available due to privacy or ethical restrictions.
