# Supplementary material for: Polarized Color Filters Using Colloidal Quantum Rod Nanocrystals for Advanced High‐Performance Displays
Source: Adv Sci (Weinh). 2025 Apr 9;12(21):2414316. doi: 10.1002/advs.202414316 (PMC12140335; doi:10.1002/advs.202414316)
Supplement: Supplementary file 1 — Supporting Information [file ADVS-12-2414316-s001.docx]

Supporting Information

**Development of Highly Efficient Polarizing Emitting Color Filters based on Colloidal Quantum Rod Nanocrystals for Advanced High-Performance Displays**

*Jianxin SONG^†,1,2^, Maksym F. Prodanov^†,1,2^, Yiyang Gao^1,2^, Chengbin Kang^1,2^, Debjyoti Bhadra^1,2^, Yuechu Cheng^1,2^, Vigneshwaran Swaminathan^1,2^, Zebin Liao^1,2^, Kumar Mallem^1,2^,* *Valerii V. Vashchenko^1,2^, Xiao Wu^4^, Xinhui Liu^4^ and Abhishek K. Srivastava*^,1,2,3^*

^1.^ State Key Laboratory of Advanced Displays and Optoelectronics Technologies, Department of Electronics and Computer Engineering, The Hong Kong University of Science and Technology, Clear Water Bay, Hong Kong, China.

^2.^ Centre for Display Research, Department of Electronics and Computer Engineering, The Hong Kong University of Science and Technology, Clear Water Bay, Hong Kong, China.

^3.^ IAS Center for Quantum Technologies, The Hong Kong University of Science and Technology, Clear Water Bay, Hong Kong, China.

^4.^ Department of Physics,

The Chinese University of Hong Kong, Hong Kong, China.

E-mail: [eeabhishek@ust.hk](mailto:eeabhishek@ust.hk)

^†^ These authors contributed equally to this work.


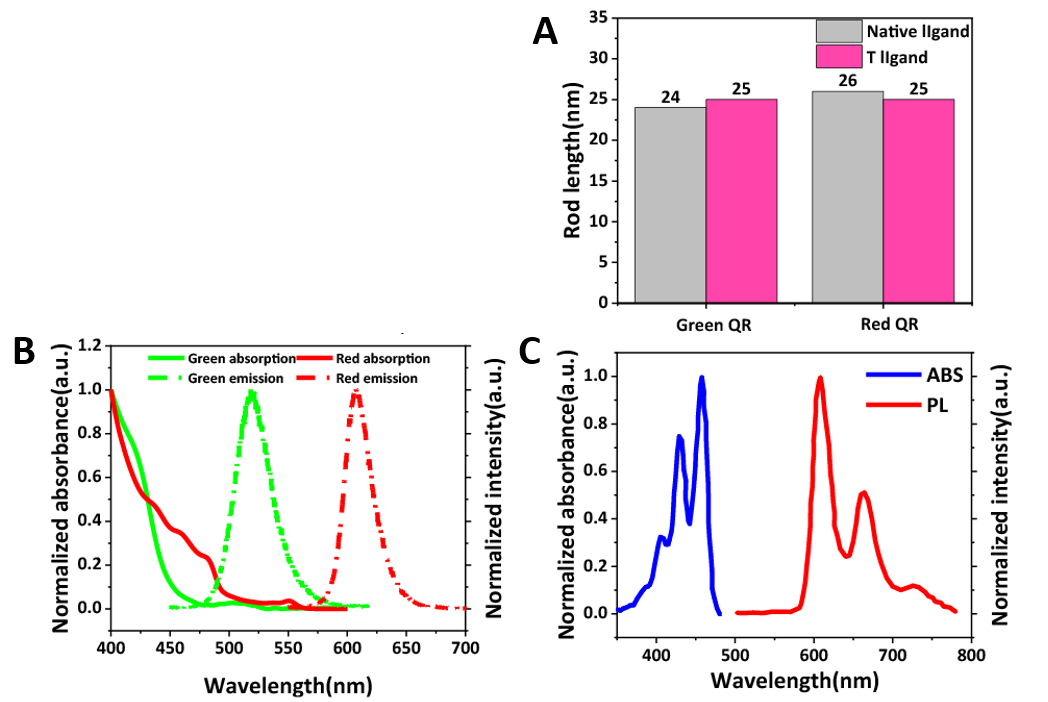


**Figure S1.** A) Quantum rod length and comparison for native ligand and T-shape ligand; Absorption and PL spectrum of B) green and red QRs and D) organic dye.

The optimization of the inkjet printing process for film fabrication can be achieved by controlling parameters such as ink viscosity (*γ*, mPas), surface tension (*σ*, m**_·_**Nm^−1^), density (*ρ*, g**_·_**cm^−3^), and nozzle diameter (*d*, μm), which govern the Ohnesorge number, defined as：

$$Z=\sqrt{\sigma\rho d}/\gamma$$

In this study, we utilized a 50% v/v CB/DCB mixed solvent to maintain the ink viscosity and surface tension within an optimal range, resulting in an Ohnesorge number (Z) closer to 14.^[38]^


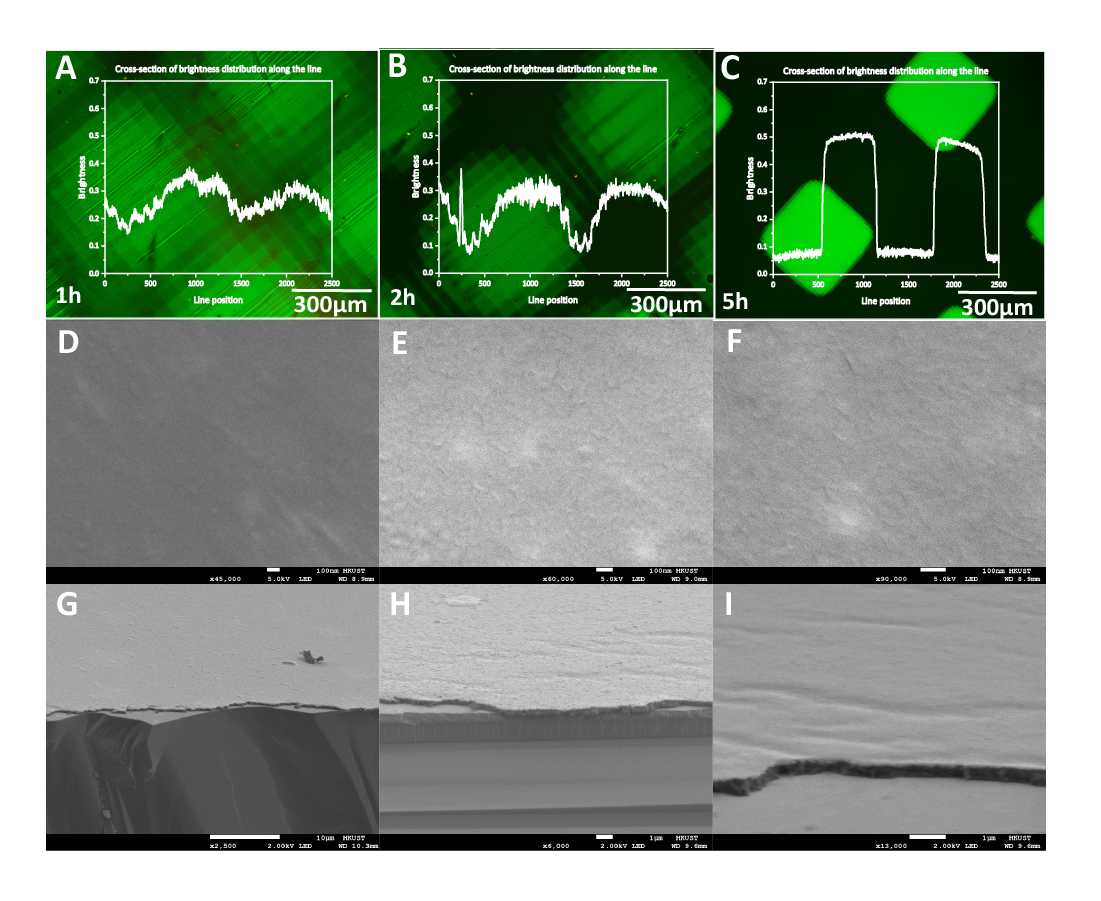


**Figure S2.** The FM image and diagonal brightness grayscale distribution curve of A)1h, B) 2h, C) 5h rinsed PLCF film. The SEM picture of surface morphology for D)×45000; E) ×60000; F) ×90000 and edge for G)×2500; H) ×6000; I) ×13000.

**
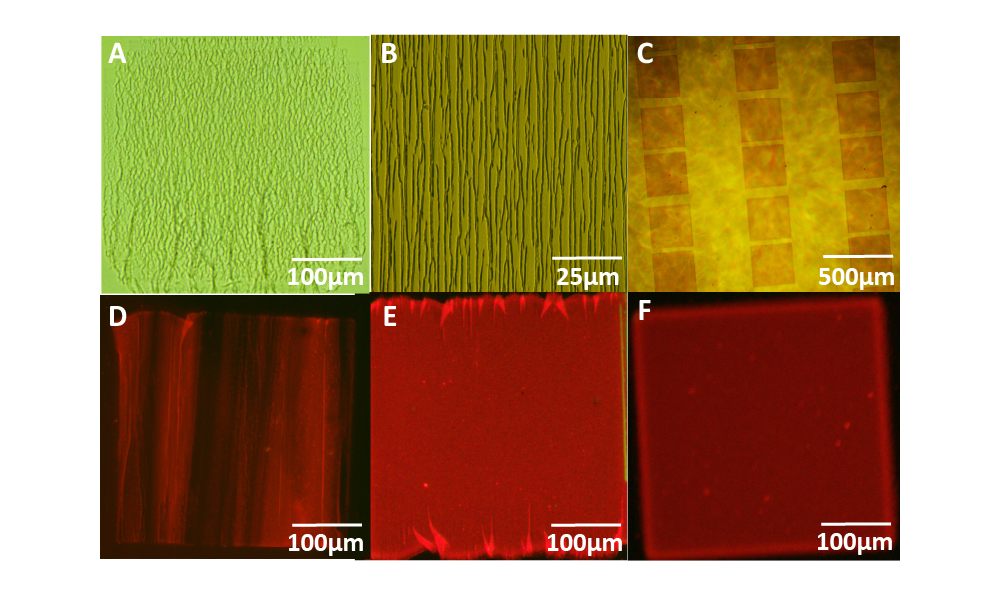
**

**Figure S3.** The FM image of fabricated CFs when A and B) LCM is not perfectly cured; D and E) too long washing time. And C and F) after the UV exposure energy and rinse time optimized


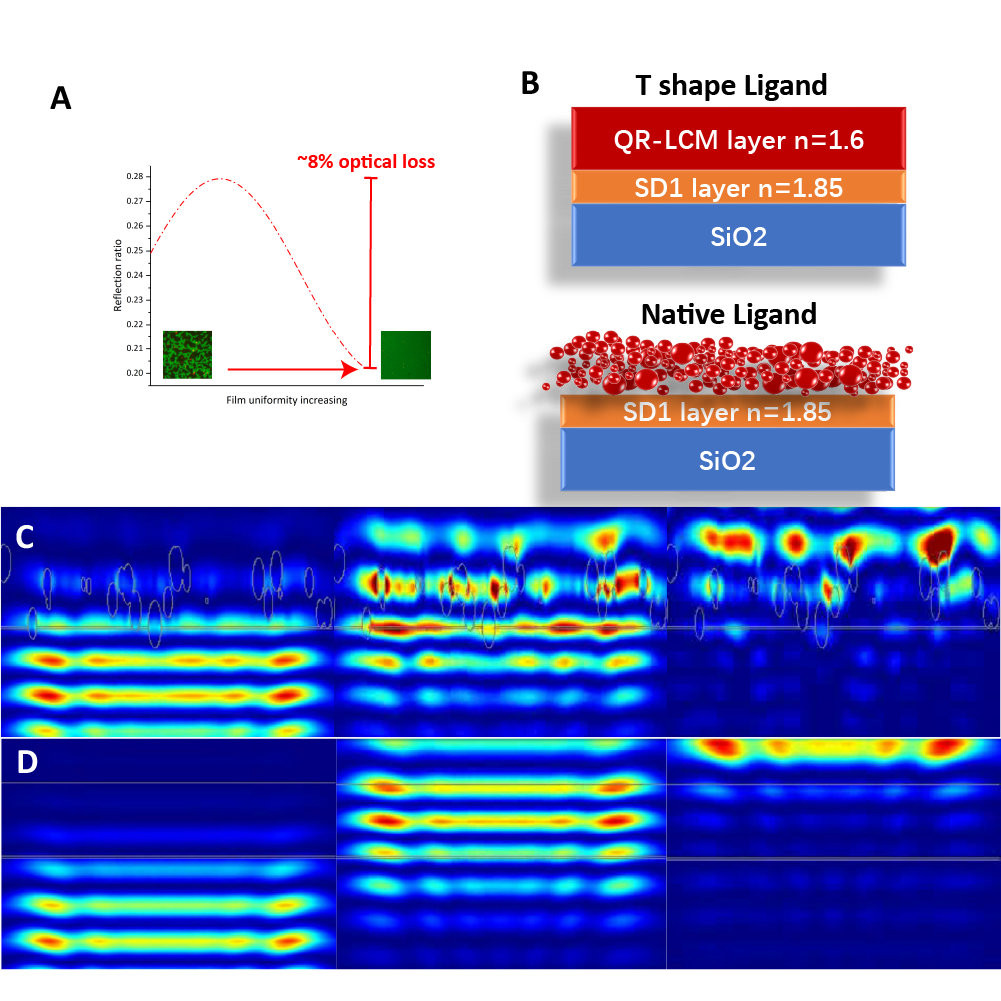


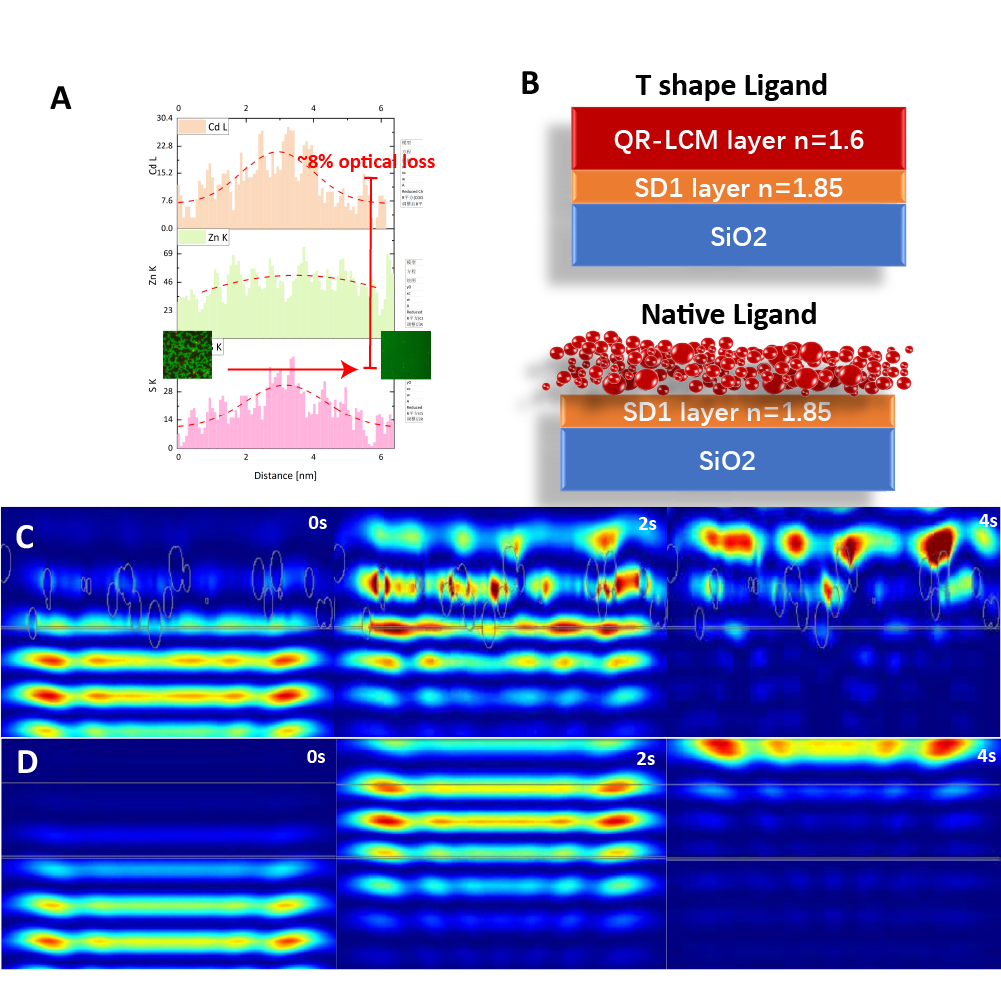


**Figure S4.** A) Reflection ratio FDTD simulation result for CFs of different morphology; B) Simulation parameters and C-D) E field distribution as the light propagating.


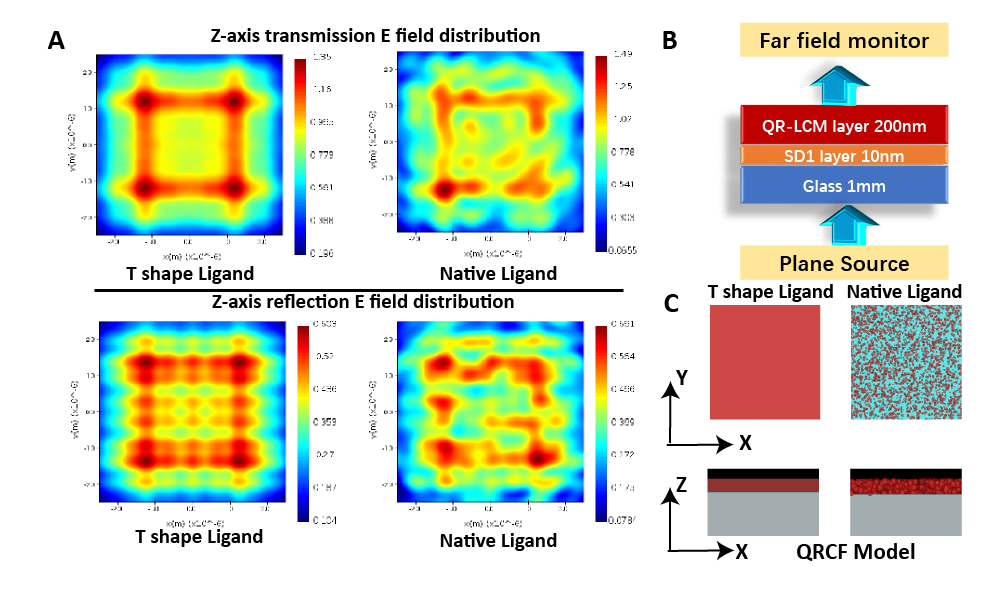


**Figure S5.** A) FDTD simulation result of different uniformity CF. B) FDTD simulation model of CF. C) And the mode difference between two kinds of ligands.


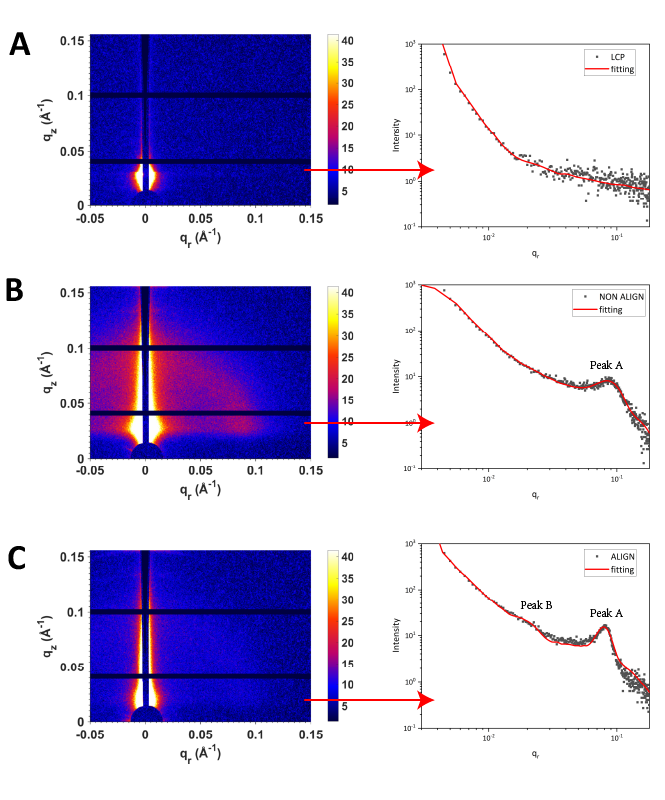


**Figure S6.** 2D GISAXS patterns and the corresponding GISAXS intensity with their fittings. A) SD1+LCP aligned on the glass substrate. B) SD1+LCP+QRs non-aligned on the glass substrate. C) SD1+LCP+QRs aligned on the glass substrate.


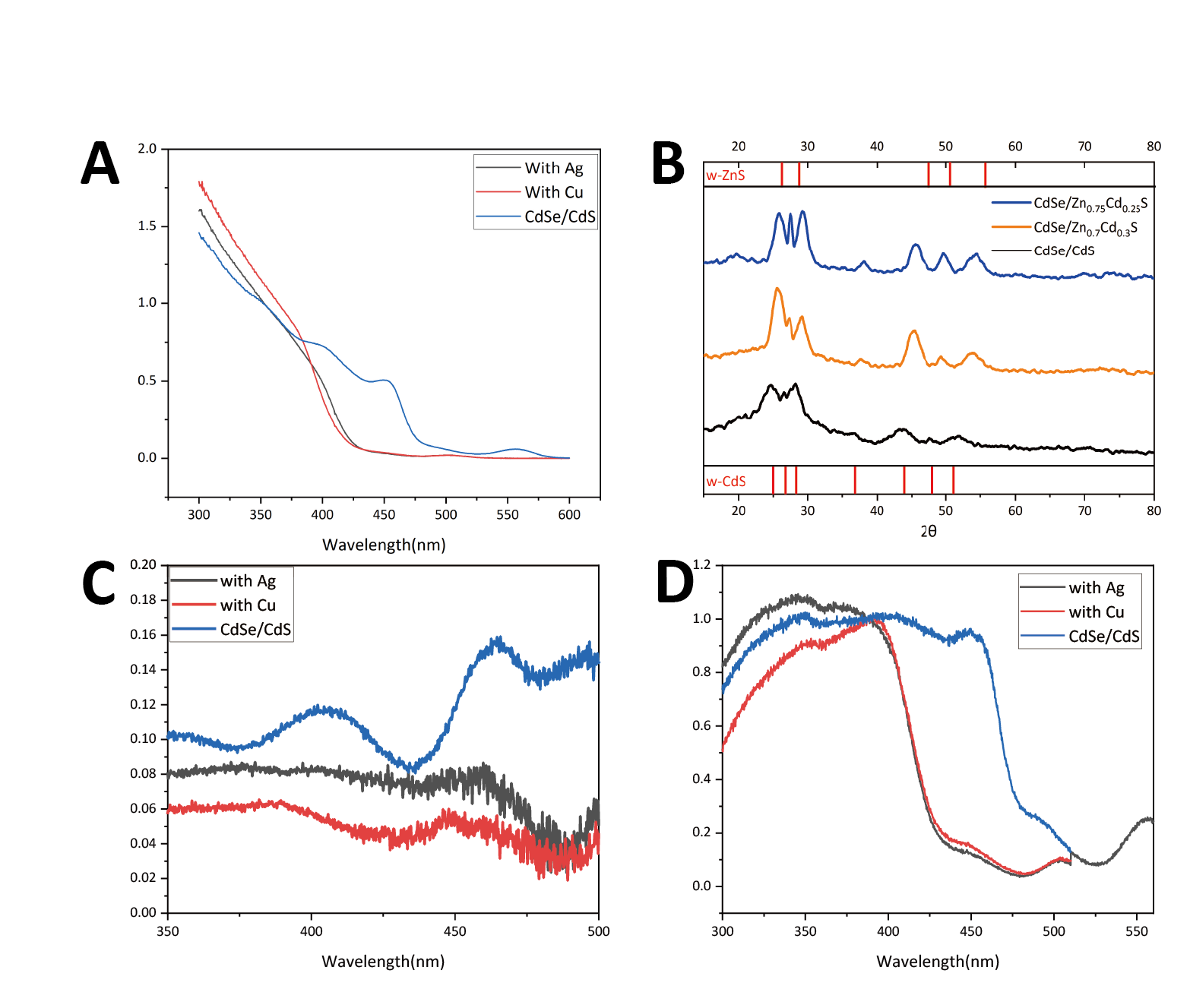


**Figure S7.** A) Absorption plot; B) X-ray diffraction patterns; C) excitation anisotropy and D) Excitation spectrum of CdSe/CdS nanorods without and with Zn doped with different elements.


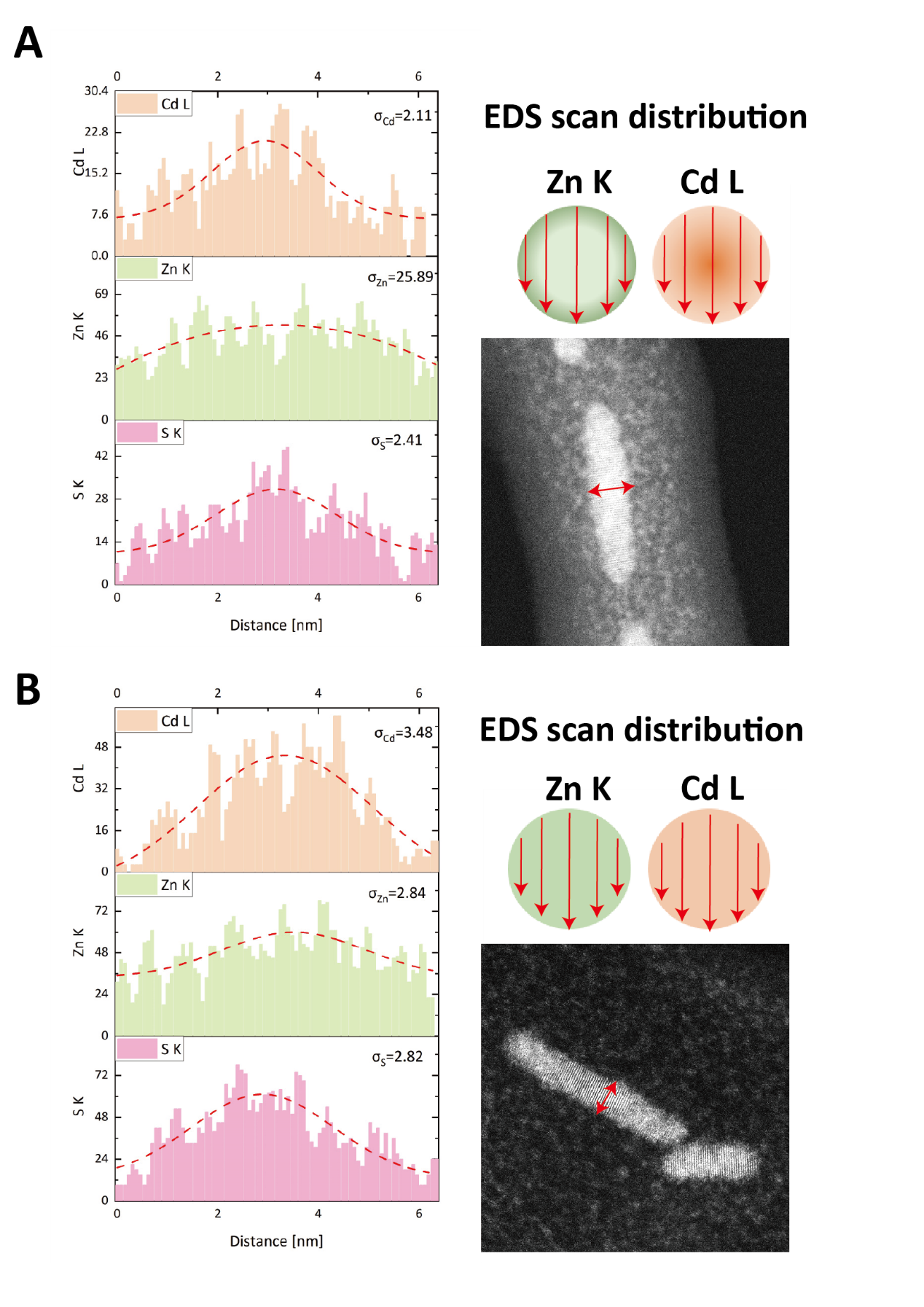


**Cu^+^**

**Ag^+^**

**Figure S8.** Element distribution of gradient alloyed QRs. A) The silver doped QRs. B) The copper doped QRs


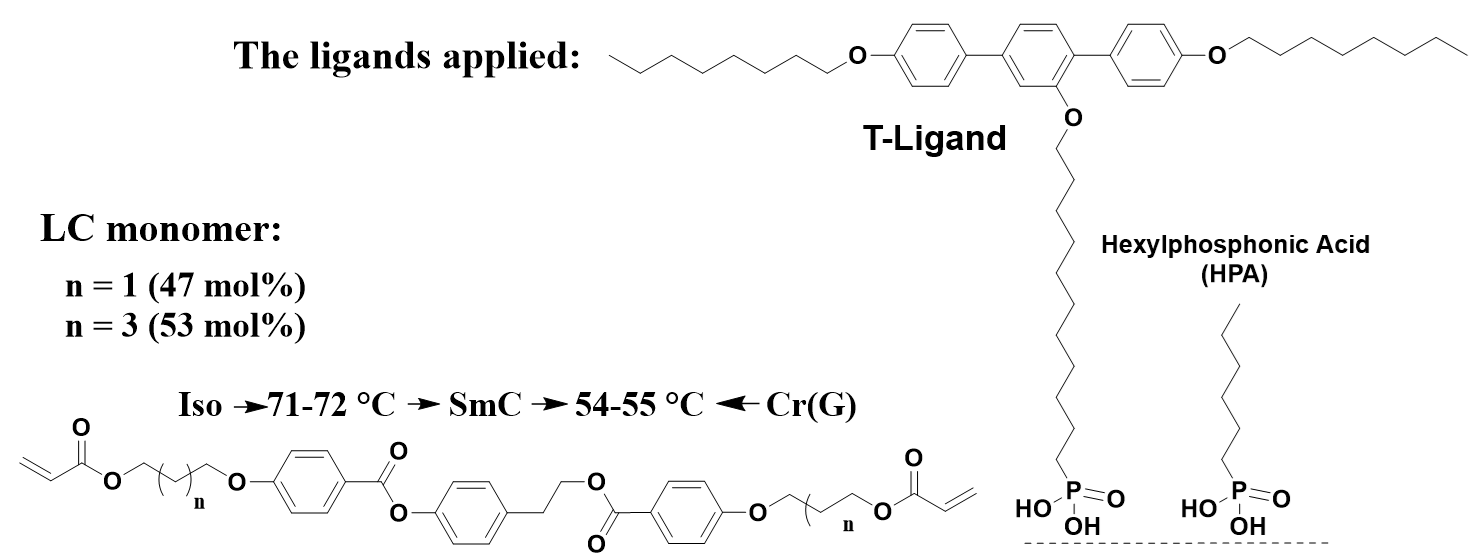


**Figure S9**. Chemical structures of the applied ligands and liquid crystal monomer with the LCM phase transitions.

**
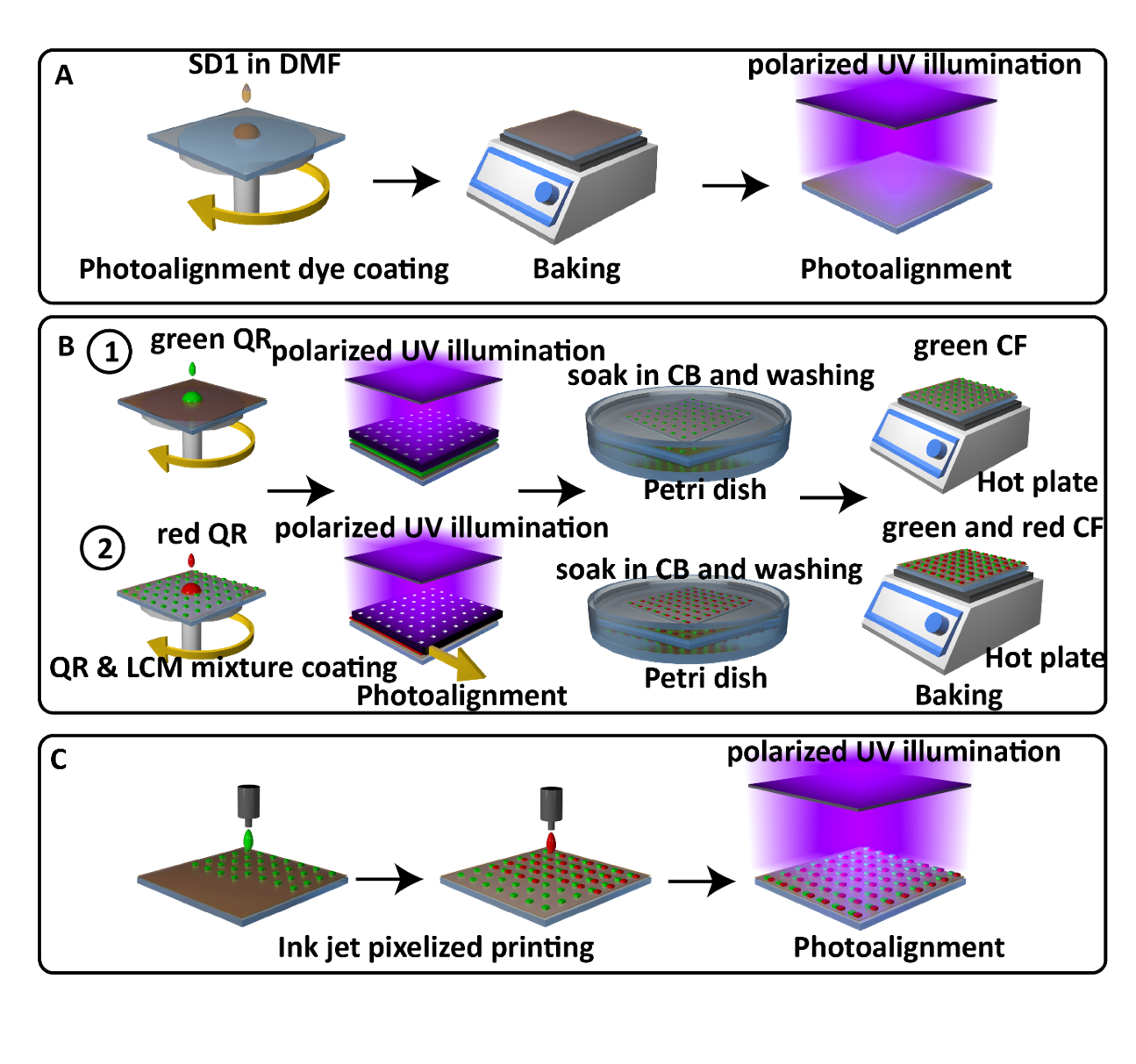
**

**Figure S10.** Schematic illustrations of the photoalignment of A) azo dye layer and B) QR and LCM mixture coating and pixelated alignment and C) pixelated ink jet printing.


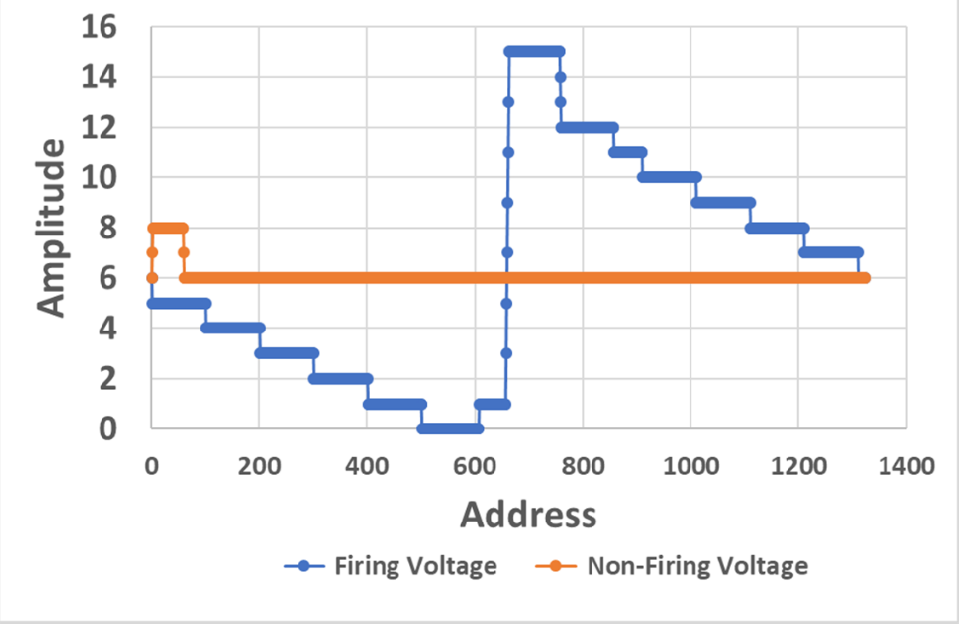


**Figure S11:** Multistep printing drive waveform

**Table S1.** Parameters of QR inks for IJP and photolithography

| **Ink composition** | **Vapor pressure (hPa)** | **Viscosity (cP)** | **Surface tension (m Nm^−1^)** |
| --- | --- | --- | --- |
| QR in 1:1 CB: DCB volume | 6.9 | 1.9 | 38 |
| QR in CB | 12 | 1.6 | 37 |

**Uniformity quantification**

Here we added a program to scan and identify the grayscale image. Applying it to the images for the films with QRs and native ligands to the T-ligands, we obtained quantitative comparison results.

The program is based on MATLAB. First, the image is converted to grayscale. The program uses the visualization technology of the three-dimensional surface map to present the smoothed grayscale image as a three-dimensional model, where the Z axis represents the grayscale intensity, thus providing an intuitive perspective for subsequent image analysis. We use the coefficient of variation (CV) to quantify the uniformity of the intensity values ​​in a selected image region. The CV is calculated as follows:

$$CV=\frac{\sigma_{std}}{\mu}$$

Which $\sigma_{std}$ is the standard derivation and μ is the mean of data. For the T-ligand PECF, the calculated CV is 0.03, indicating a high degree of uniformity in the intensity values ​​in this region. In contrast, the CV for the native ligand is 0.2, indicating significantly greater variability and less uniformity across its surface compared to the ligand PECF.


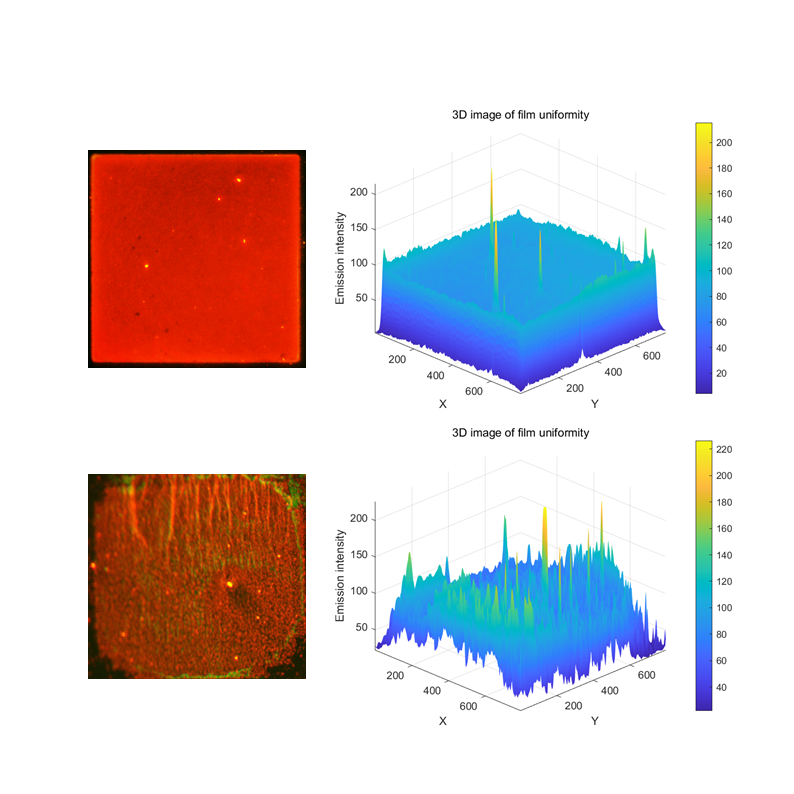


**Figure S12:** Uniformity quantification processing result images

**Figure S13:** PECF film quantum yield washing stability during the fabrication process

# Color gamut calculation.

We measured the device in different switch on states (R/G/B) using an integrating sphere and a spectrometer, entered the obtained spectral data into the program and performed calculations.

The calculation details of the program are as follows:


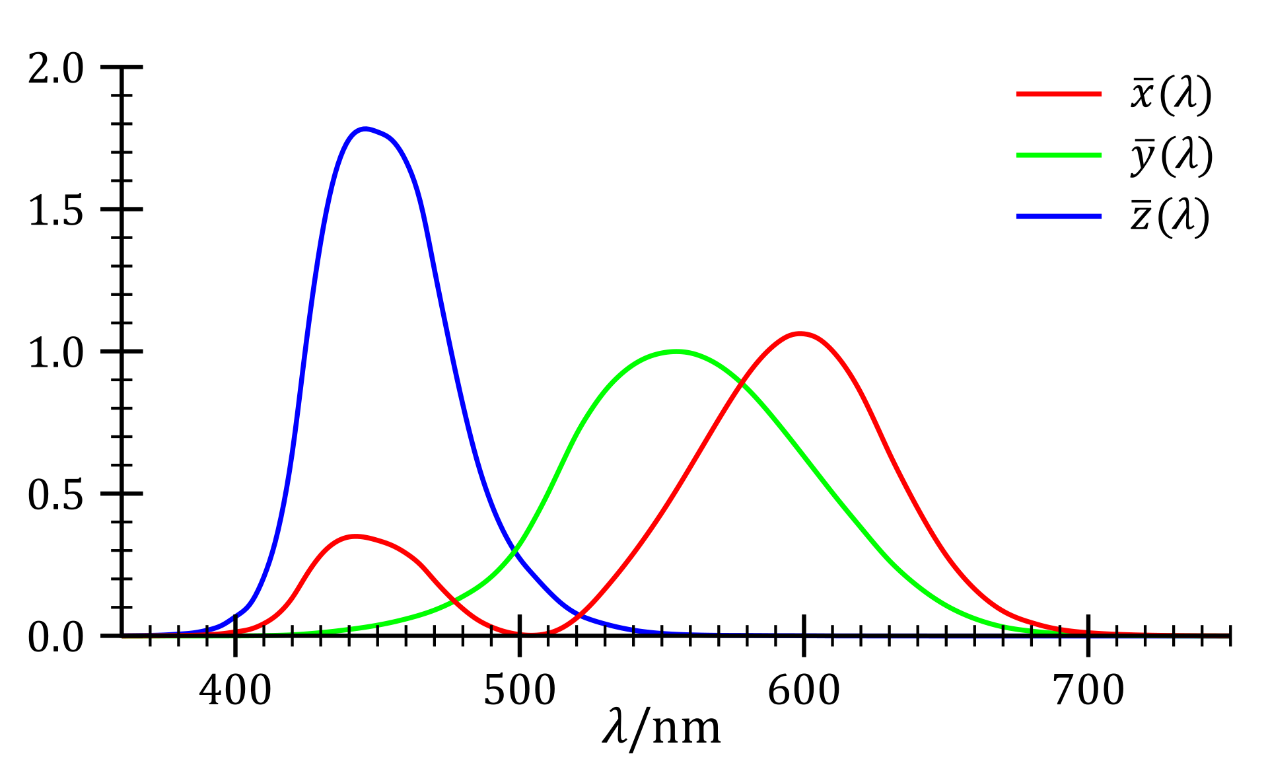


**Figure S14:** The CIE XYZ standard observer color matching functions

We first convert the spectrum into X, Y, Z according to the CIE standard three primary color stimulus values. The calculation follows the following formula:

$$X=\frac{K}{N}\int S\left( \lambda\right)I\left( \lambda\right)\bar{x}\left( \lambda\right)d\lambda$$

$$Y=\frac{K}{N}\int S\left( \lambda\right)I\left( \lambda\right)\bar{y}\left( \lambda\right)d\lambda$$

$$Z=\frac{K}{N}\int S\left( \lambda\right)I\left( \lambda\right)\bar{z}\left( \lambda\right)d\lambda$$

$$N=\int I\left( \lambda\right)\bar{y}\left( \lambda\right)d\lambda$$

In which X, Y, and Z represent three stimulus values, S(λ) is the transmittance, and I(λ) is the light source power. Multiply them to get the light power. Then, by integrating the light power over the entire wavelength, we get the X, Y, and Z three stimulus values, and finally calculate the x, y, and z coordinates through the ratio between X, Y, Z and (X+Y+Z).

Through the x,y and z coordinates from three spectrums(R/G/B states), we can get the color triangle and relative area which correspond to the color gamut.

# Effect of ligand exchange on QRs properties


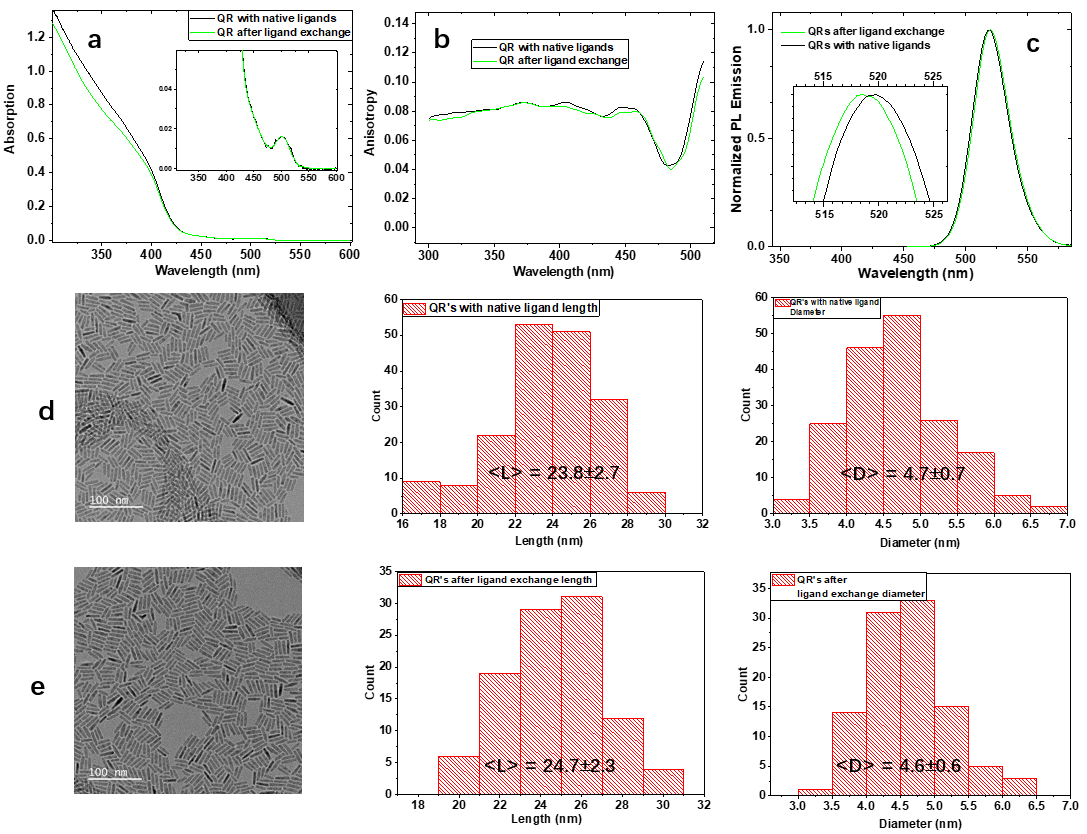


**Figure S15**. (a) Absorption, (b) excitation anisotropy and (c,d) TEM data for QR’s before and after the ligand exchange with mixture of T-shape ligand and hexylphosphonic acid.

We observed only marginal changes in the material properties. Specifically, absorption shows a slight decrease in the UV range (Figure 15a), which may indicate minor removal of surface Zn/Cd atoms together with the initial native ligand molecules, during their replacement with new phosphonic acid ligands. However, no significant changes were detected in the polarization properties of the material, as confirmed by measurements of the QRs’ excitation anisotropy (Figure 15b). Furthermore, the PLQY of the material remains unaffected by the ligand exchange, maintaining ~98% for both the initial and ligand-exchanged QRs. TEM results also confirm consistent nanoparticle shape and size, with the observed variations being within the experimental error margin (Figure 15d,e).

The consistency of the material properties is attributed to the mild ligand exchange conditions (T < 160°C) and the same chemical nature of the anchoring groups in both the native and new ligands (phosphonic acids). This prevents significant changes to the QR surface properties and the formation of additional surface defects. Additionally, the aromatic core of the T-shaped ligand does not significantly impact the polarization or absorption properties, as the amount of this ligand is very low compared to the hexylphosphonic acid co-ligand (TL:HPA = 1:9). This ratio is dictated by the need for parallel surface alignment of the T-ligand with minimal TL-TL molecular interaction for further efficient alignment of the nanorods within LC polymer.


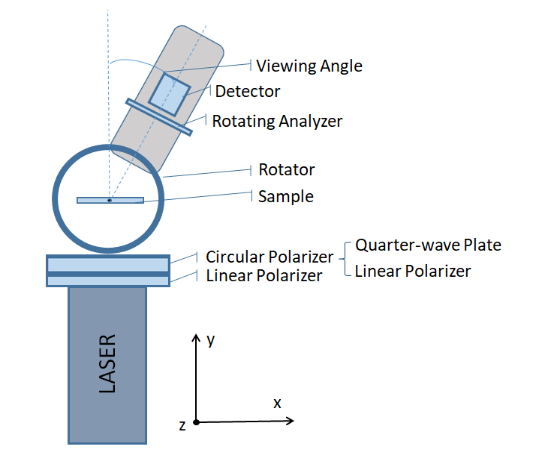

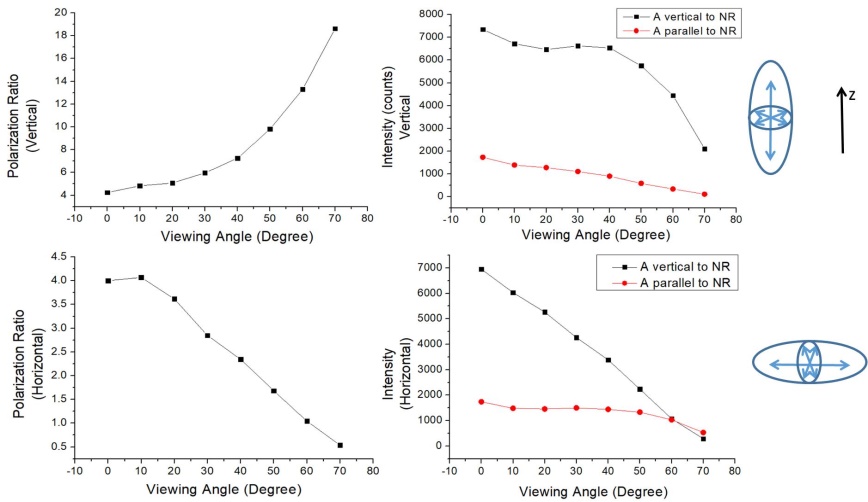


(a)

(b)

**Figure S16**. (a) Schematic diagram of the experimental setup. (b) Experiment results. Angular dependent polarized emission component vertical and parallel to the NR alignment direction and angular dependent polarization ratio
